# Supplementary material for: Effects of digital devices and online learning on computer vision syndrome in students during the COVID-19 era: an online questionnaire study
Source: BMJ Paediatr Open. 2022 Jun 9;6(1):e001429. doi: 10.1136/bmjpo-2022-001429 (PMC9184992; doi:10.1136/bmjpo-2022-001429)
Supplement: Supplementary data [file bmjpo-2022-001429supp001.pdf]

Effects of digital devices and online learning on computer vision syndrome in students during the COVID-19 era

Survey

The purpose of this survey: This survey aims to gather information from students of different year groups about what visual symptoms they experience and how well they protect their eyes during the period of online learning, since it is compulsory for students to sit in front of their screens for a long period of time. This results of this survey will then be analyzed, conclusion will be drawn and the results will then be incorporated as one of the research findings.

General overview of the survey:
First section of the survey - 8 questions
Second section of the survey - 8 questions
Third section of the survey - 18-36 questions

It will take approximately 2 minutes to fill out the survey!

Please note that the survey will be anonymous. The information will be kept confidential and will only be used for this research.

Informed consent

1. Do you voluntarily agree to complete this survey?
   - I voluntarily agreed to complete this survey

Section 1 of the survey: General information

1. Age

   ____
2. Gender
   - Male
   - Female
3. Grade level
   - Grade 1-3/Year 2-4
   - Grade 4-6/Year 5-7
   - Grade 7-9/Year 8-10
   - Grade 10-12/Year 11-13
4. Before the period of online learning, how many hours per day do you spend on average using electronic devices?
   - 0 hour
   - 1 hour
   - 2 hours
   - 3 hours
   - 4 hours
   - 5 hours
   - 6 hours
   - 7 hours

- 8 hours
- 9 hours
- 10 hours
- 11 hours
- 12 hours
- Other…………….

5. Before the period of online learning, what electronic devices did you use most often?
   - Tablet (iPad)
   - Mobile Phone
   - Computer/Desktop
   - Laptop
   - Television
   - Other
6. Do you wear any of these? (Can tick more than one box)
   - Glasses for short-sightedness
   - Glasses for long-sightedness
   - Contact lenses for short-sightedness
   - Contact lenses for long-sightedness

7. How often do you have an eye check-up?
   - Never
   - Once every two years
   - Once a year
   - Twice a year
   - Other

Section 2 of the Survey

7. What electronic devices do you use for online learning?
   - Tablet (iPad)
   - Mobile Phone
   - Computer/Desktop
   - Laptop
   - Television
   - Other
8. How many hours per day do you spent learning online?
   - 1 hour
   - 2 hours
   - 3 hours
   - 4 hours
   - 5 hours
   - 6 hours
   - 7 hours
   - 8 hours
   - 9 hours
   - 10 hours
   - 11 hours

- 12 hours
- Other…………….

9. What is the environment of your room when you learn online? Do you use a fan or an air conditioner?
   - Fan
   - Air conditioner
   - Other
10. Do you use any equipment to help protect your eyes?
    - Glasses
    - Films for computer/tablets/phone screens
11. How often do you give your eyes a rest during online classes?
    - Never
    - Every 15 minutes
    - Every 30 minutes
    - Every 45 minutes
    - Every 1 hour
    - Every 2 hours
    - Other
12. What do you do when you rest your eyes?
    - Close your eyes
    - Look out of the window as far as you could
    - Play games on your device
    - Sleep
    - Artificial tear
    - Other
13. Apart from using electronic devices for online learning, how much time do you spend using electronic devices for other activities?
    - 1 hour
    - 2 hours
    - 3 hours
    - 4 hours
    - 5 hours
    - 6 hours
    - 7 hours
    - 8 hours
    - 9 hours
    - 10 hours
    - 11 hours
    - 12 hours
    - Other…………….
14. How far apart do you position your devices from your eyes?
    - 20-40 cm
    - 40-60 cm
    - 60-80 cm
    - 80-100 cm
    - more than 100 cm

Section 3 of the survey:
This section will be the computer vision syndrome questionnaire.

The first question will be related to the frequency of the symptom, how often the symptom occurs:
Never = the symptom does not occur at all
Occasionally = Sporadic episodes or once a week
Often or always = 2 or 3 times a week or almost everyday

NOTE: If you have answered "Never" for the first question, you will not be asked about the intensity, but if you answered "Occasionally" or "Often or always", you will be required to answer the question about intensity.

The second question will be related to the intensity of the symptom. You are required to choose between "moderate" or "Intense" for each of the symptoms.

15. When you learn online, do you experience the symptom of eye burning?
    - Never (the symptom does not occur)
    - Occasionally (once a week)
    - Often or always (2 or 3 times a week or almost everyday)
16. What is the intensity of the symptom? (If you answered never, do not answer this question)
    - Moderate
    - Intense
17. When you learn online, do you experience the symptom of eye itching?
    - Never (the symptom does not occur)
    - Occasionally (once a week)
    - Often or always (2 or 3 times a week or almost everyday)
18. What is the intensity of the symptom? (If you answered never, do not answer this question)
    - Moderate
    - Intense
19. When you learn online, have you ever feel like there’s foreign body in your eyes?
    - Never (the symptom does not occur)
    - Occasionally (once a week)
    - Often or always (2 or 3 times a week or almost everyday)
20. What is the intensity of the symptom? (If you answered never, do not answer this question)
    - Moderate
    - Intense
21. When you learn online, do you experience the symptom of tearing?
    - Never (the symptom does not occur)
    - Occasionally (once a week)
    - Often or always (2 or 3 times a week or almost everyday)
22. What is the intensity of the symptom? (If you answered never, do not answer this question)
    - Moderate

- Intense

23. When you learn online, do you experience the symptom of excessive blinking?
    - Never (the symptom does not occur)
    - Occasionally (once a week)
    - Often or always (2 or 3 times a week or almost everyday)
24. What is the intensity of the symptom? (If you answered never, do not answer this question)
    - Moderate
    - Intense
25. When you learn online, do you experience the symptom of eye redness?
    - Never (the symptom does not occur)
    - Occasionally (once a week)
    - Often or always (2 or 3 times a week or almost everyday)
26. What is the intensity of the symptom? (If you answered never, do not answer this question)
    - Moderate
    - Intense
27. When you learn online, do you experience the symptom of eye pain?
    - Never (the symptom does not occur)
    - Occasionally (once a week)
    - Often or always (2 or 3 times a week or almost everyday)
28. What is the intensity of the symptom? (If you answered never, do not answer this question)
    - Moderate
    - Intense
29. When you learn online, do you experience the symptom of heavy eyelids?
    - Never (the symptom does not occur)
    - Occasionally (once a week)
    - Often or always (2 or 3 times a week or almost everyday)
30. What is the intensity of the symptom? (If you answered never, do not answer this question)
    - Moderate
    - Intense
31. When you learn online, do you experience the symptom of eye dryness?
    - Never (the symptom does not occur)
    - Occasionally (once a week)
    - Often or always (2 or 3 times a week or almost everyday)
32. What is the intensity of the symptom? (If you answered never, do not answer this question)
    - Moderate
    - Intense
33. When you learn online, do you experience the symptom of blurred vision?
    - Never (the symptom does not occur)
    - Occasionally (once a week)
    - Often or always (2 or 3 times a week or almost everyday)
34. What is the intensity of the symptom? (If you answered never, do not answer this question)

- Moderate
- Intense

35. When you learn online, do you experience the symptom of double vision?
    - Never (the symptom does not occur)
    - Occasionally (once a week)
    - Often or always (2 or 3 times a week or almost everyday)
36. What is the intensity of the symptom? (If you answered never, do not answer this question)
    - Moderate
    - Intense
37. When you learn online, how often do you experience difficulties focusing near objects?
    - Never (the symptom does not occur)
    - Occasionally (once a week)
    - Often or always (2 or 3 times a week or almost everyday)
38. What is the intensity of the symptom? (If you answered never, do not answer this question)
    - Moderate
    - Intense
39. When you learn online, do you experience the symptom of increased sensitivity to light?
    - Never (the symptom does not occur)
    - Occasionally (once a week)
    - Often or always (2 or 3 times a week or almost everyday)
40. What is the intensity of the symptom? (If you answered never, do not answer this question)
    - Moderate
    - Intense
41. When you learn online, do you experience the symptom of colored halos around objects?
    - Never (the symptom does not occur)
    - Occasionally (once a week)
    - Often or always (2 or 3 times a week or almost everyday)
42. What is the intensity of the symptom? (If you answered never, do not answer this question)
    - Moderate
    - Intense
43. When you learn online, do you experience the feeling that sight is worsening?
    - Never (the symptom does not occur)
    - Occasionally (once a week)
    - Often or always (2 or 3 times a week or almost everyday)
44. What is the intensity of the symptom? (If you answered never, do not answer this question)
    - Moderate
    - Intense
45. When you learn online, how often do you have a headache?
    - Never (the symptom does not occur)

- Occasionally (once a week)
- Often or always (2 or 3 times a week or almost everyday)

46. What is the intensity of the symptom? (If you answered never, do not answer this question)
    - Moderate
    - Intense
47. When you learn online, how often do you experience back pain?
    - Never (the symptom does not occur)
    - Occasionally (once a week)
    - Often or always (2 or 3 times a week or almost everyday)
48. What is the intensity of the symptom? (If you answered never, do not answer this question)
    - Moderate
    - Intense
49. When you learn online, how often do you experience neck pain?
    - Never (the symptom does not occur)
    - Occasionally (once a week)
    - Often or always (2 or 3 times a week or almost everyday)
50. What is the intensity of the symptom? (If you answered never, do not answer this question)
    - Never (the symptom does not occur)
    - Occasionally (once a week)
    - Often or always (2 or 3 times a week or almost everyday)

Thank you for completing this survey!
