# Supplementary material for: Effects of digital devices and online learning on computer vision syndrome in students during the COVID-19 era: an online questionnaire study
Source: BMJ Paediatr Open. 2022 Jun 9;6(1):e001429. doi: 10.1136/bmjpo-2022-001429 (PMC9184992; doi:10.1136/bmjpo-2022-001429)
Supplement: Author's manuscript [file bmjpo-2022-001429.draft_revisions.pdf]

BMJ Paediatrics Open

BMJ Paediatrics Open is committed to open peer review. As part of this commitment we make the peer review history of every article we publish publicly available.

When an article is published we post the peer reviewers’ comments and the authors’ responses online. We also post the versions of the paper that were used during peer review. These are the versions that the peer review comments apply to.

The versions of the paper that follow are the versions that were submitted during the peer review process. They are not the versions of record or the final published versions. They should not be cited or distributed as the published version of this manuscript.

BMJ Paediatrics Open is an open access journal and the full, final, typeset and author-corrected version of record of the manuscript is available on our site with no access controls, subscription charges or pay-per-view fees (http://bmjpaedsopen.bmj.com).

If you have any questions on BMJ Paediatrics Open’s open peer review process please email info.bmjpo@bmj.com

BMJ Paediatrics Open

Effects of digital devices and online learning on computer vision syndrome in students during the COVID-19 era

Journal:	*BMJ Paediatrics Open*
Manuscript ID	bmjpo-2022-001429
Article Type:	Original research
Date Submitted by the Author:	27-Jan-2022
Complete List of Authors:	Seresirikachorn, Kasem; Thammasat University Sirindhorn International Institute of Technology<br>Thiamthat, Warakorn; Rajavithi Hospital, Department of Ophthalmology, College of Medicine, Rangsit University<br>Sriyuttagrai, Wararee; Rajavithi Hospital, Department of Ophthalmology, College of Medicine, Rangsit University<br>Soonthornworasiri, Ngamphol; Mahidol University, Department of Tropical Hygiene<br>Singhanetr, Panisa; Mettapracharak Hospital, Department of Ophthalmology<br>Yudtanahiran, Narata; Anglo Singapore International School<br>Theeramunkong, Thanaruk; Thammasat University Sirindhorn International Institute of Technology; Royal Society of Thailand
Keywords:	COVID-19, Adolescent Health, Information Technology, Ophthalmology, Epidemiology

SCHOLARONE™
Manuscripts

I, the Submitting Author has the right to grant and does grant on behalf of all authors of the Work (as defined in the below author licence), an exclusive licence and/or a non-exclusive licence for contributions from authors who are: i) UK Crown employees; ii) where BMJ has agreed a CC-BY licence shall apply, and/or iii) in accordance with the terms applicable for US Federal Government officers or employees acting as part of their official duties; on a worldwide, perpetual, irrevocable, royalty-free basis to BMJ Publishing Group Ltd ("BMJ") its licensees and where the relevant Journal is co-owned by BMJ to the co-owners of the Journal, to publish the Work in this journal and any other BMJ products and to exploit all rights, as set out in our licence.

The Submitting Author accepts and understands that any supply made under these terms is made by BMJ to the Submitting Author unless you are acting as an employee on behalf of your employer or a postgraduate student of an affiliated institution which is paying any applicable article publishing charge ("APC") for Open Access articles. Where the Submitting Author wishes to make the Work available on an Open Access basis (and intends to pay the relevant APC), the terms of reuse of such Open Access shall be governed by a Creative Commons licence – details of these licences and which Creative Commons licence will apply to this Work are set out in our licence referred to above.

Other than as permitted in any relevant BMJ Author's Self Archiving Policies, I confirm this Work has not been accepted for publication elsewhere, is not being considered for publication elsewhere and does not duplicate material already published. I confirm all authors consent to publication of this Work and authorise the granting of this licence.

Confidential: For Review Only

Effects of digital devices and online learning on computer vision syndrome in students during the COVID-19 era

Kasem Seresirikachorn, MD [1]
Warakorn Thiamthat, MD [2]
Wararee Sriyuttagrai, MD [2]
Ngamphol Soonthornworasiri, PhD [3]
Panisa Singhanetr, MD [4]
Narata Yudtanahiran [5]
Thanaruk Theeramunkong, PhD [1,6]

[1] Sirindhorn International Institute of Technology, Thammasat University, Pathumthani, Thailand.
[2] Department of Ophthalmology, College of Medicine, Rangsit University, Rajavithi Hospital, Bangkok, Thailand.
[3] Department of Tropical Hygiene, Faculty of Tropical Medicine, Mahidol University, Bangkok, Thailand.
[4] Department of Ophthalmology, Mettapracharak Hospital, Nakhon Pathom, Thailand.
[5] Anglo Singapore International School, Bangkok, Thailand
[6] The Royal Society of Thailand, Bangkok, Thailand.

Running title: Effects of digital devices and online learning on computer vision syndrome in students during the COVID-19 era

None of the authors have any conflict of interest to disclose

Correspondence: Thanaruk Theeramunkong PhD,
Sirindhorn International Institute of Technology, Thammasat University,
131 Moo5, Tiwanont Road,
Bangkadi, Muang
Pathumthani 12000, Thailand.
Tel: 66 2-501-3505 ext 6018
Fax: 66 2-501-3505 ext 6014
Email: thanaruk@siit.tu.ac.th

Confidential: For Review Only

Effects of digital devices and online learning on computer vision syndrome in students during the COVID-19 era

Abstract (274 words)

Purpose : Computer vision syndrome (CVS) is a group of eye and vision-related problems that result from prolonged digital device use. This study aims to assess the prevalence and risk factors of CVS among students during lockdown from the COVID-19 pandemic.

Methods: A cross-sectional, online, questionnaire-based study done among high school students in Thailand.

Results: A total of 2,476 students were included in this study, with mean age of 15.52±1.66 years. The number of hours of digital device use per day (10.53 ± 2.99) increased during the COVID-19 pandemic compared to before the pandemic (6.13 ± 2.8). The mean number of hours of online learning was 7.03 ± 2.06 hours per day. CVS was found in 70.1% of students with severity correlating with number of hours of online learning, and total number of hours of digital device usage (P<0.001). In contrast, age was inversely correlated with the severity of CVS (P<0.001). Multivariate analysis revealed significant risk factors of CVS including, age ≤ 15 years (adjusted odds ratio (AOR)=2.17), overall digital device usage > 6 hours per day (AOR=1.91), online learning > 5 hours per day (AOR=4.99), multiple digital device usage (AOR=2.15), refractive errors (AOR=2.89), presence of back pain (AOR=2.06), and presence of neck pain (AOR=2.36).

Conclusions: The number of hours of digital device usage increased during lockdown. Over 70% of children have CVS. Multiple risk factors including hours of digital device usage, hours of online learning, ergonomics, and refractive error should be adjusted to decreased the risk of CVS. Online learning will remain, along with CVS after this pandemic. We hope our study could be taken into account in remodeling our education system accordingly.

Keywords: Coronavirus disease (COVID-19), computer vision syndrome (CVS), digital eye strain, severe acute respiratory syndrome coronavirus 2 (SARS-CoV-2)

Confidential: For Review Only

Key Messages

What is known about the subject ?

- Computer vision syndrome is a group of vision-related problems caused by prolonged use of digital devices.
- Learning has shifted from in schools to online during the COVID-19 pandemic.

What this study adds ?

- Digital device use has increased 4 hours per day during the COVID-19 pandemic with average online learning of 7.03 ± 2.06 hours per day.
- Computer vision syndrome was found in 70.1% of students.
- Risk factors of CVS include, digital device usage > 6 hours/day, multiple digital device usage, refractive errors, and presence of back and neck pain.

1. Background

In 2019, there was a report from Wuhan Municipal Health Commission about a cluster of pneumonia in Wuhan, China. (1-3) What started as an outbreak in China is now a global crisis. On March 11, 2020, WHO announced that COVID-19 can be classified as a pandemic. (4) Suppression aims to slow down epidemic growth by reducing the number of cases and human to human transmission with social distancing and closure of schools and universities. (5, 6) UNESCO reported 1.37 billion students from over 130 countries affected by these interventions. For Thailand, UNESCO reported the total duration of school closure of 42 weeks. (7)

Many schools shifted from classroom-based learning to online learning in order to continue with education. A learning system that is based on teaching with the help of electronic sources, or e-learning is a suitable option for all levels of education. Therefore, the benefits of digital devices cannot be denied. (8) Easily accessible learning systems also has it's downside. Up to 54% of parents of children aged 5 to 15 years reported up to 5 additional hours online on average.(9) Moderate use of screens (4 hours/day) was associated with lower psychological well-being including, less curiosity, lower self-control, distractibility, and inability to finish tasks. (10)

Rapid advancement in technology has lead digital devices to become a big part of our daily lives, some more than others. Prolonged screen time can produce physical

Confidential: For Review Only

discomfort known as digital eye strain (DES), or computer vision syndrome (CVS), which may be expressed through various symptoms such as, eyestrain, headache, blurred vision, and dry eye symptoms.(11) This study aims to assess the prevalence and risk factors of computer vision syndrome among high school students in Bangkok during lockdown from the COVID-19 pandemic.

2. Materials and Methods

This cross-sectional, online, questionnaire-based study was approved by the institutional review boards of Rajavithi Hospital, Thailand, and was conducted in accordance with the Declaration of Helsinki. The online questionnaire was sent to high school students from grade 4 to grade 12 electronically. Before answering the questionnaire, all participants were informed about the purpose, method, and confidentiality of data on the cover letter of the questionnaire. All participants have to sign and accept the informed consent before continuing with the survey. Data was collected between August 16, 2021 and August 31, 2021 (15 days), during school closures in accordance with the COVID-19 lockdown policy.

Online questionnaire

The questionnaire consisted of 4 parts including, demographic data and electronic device usage before the pandemic, online learning behaviors, Computer Vision Syndrome Questionnaire (CVS-Q), and a poster providing students with information on the proper use of electronic devices. (Supplementary 1)

The CVS-Q was developed by Segui et al. (12) This questionnaire is comprised of 16 eye symptoms. High school students are required to report the frequency and intensity of each eye symptom. For each symptom, frequency score is multiplied by intensity score and adding all of the scores for each symptom together. For the person to be considered having computer vision syndrome, he or she must have a total score of greater than or equal to six. The severity was divided into mild, moderate, and severe, with scores 6-12, 13-18, and 19 or over respectively.

Statistical Analysis

Confidential: For Review Only

All the data from the electronic survey was analyzed with SPSS 16.0 for Windows (SPSS Inc., Chicago, IL, USA). Descriptive statistics were used for categorical data. Continuous data was reported using mean, median, and standard deviation (SD). Paired-T test was used to compare the number of hours of digital device used during the COVID pandemic compared to before the pandemic. One-way ANOVA was used to compare hours of online learning, total hours of digital device usage, age of students, and severity of CVS. Risk factors associated with CVS were analyzed by univariate and multivariate logistic regression to identify independent risk factors of CVS by calculating the odds ratio (OR) and their corresponding 95% confidence interval (CI). A P-value < 0.05 was considered statistically significant.

3. Results

Baseline Characteristics

A total of 2,476 students completed the online survey. Demographic data and electronic device usage before the pandemic is shown in Table 1. The mean age was 15.52 ± 1.66 years with female predominance (64.9%). The majority of students were in grades 10-12 (68%). Around 40% of the students use glasses or contact lenses to correct for their refractive errors. Mobile phones were the most used digital device before the COVID-19 pandemic at 54.2%. A quarter of students spend around 5-6 hours of screen time before the lockdown.

Digital Device Usage During the COVID-19 pandemic

The average number of hours spent per day using digital devices before the pandemic was 6.13 ± 2.8, which increased to an average of 10.53 ± 2.99 during lockdown (P< 0.001). More than half of the students use digital devices for 9-12 hours/day (mean = 7.03 ± 2.06 hours/day). Forty percent of the students use multiple devices when learning online, with mobile phones being the most used device. Most students do their online studying in a fan environment. Over 80% of students use some sort of protective equipment including, blue-coated glasses, or protective films on their digital devices. Symptoms of back pain and neck pain were present in around 75.9% and 68.1% of students during lockdown, respectively. (Table 2)

Confidential: For Review Only

Computer Vision Syndrome Questionnaire

The median score of CVS-Q was 11 (range 0-64). Among all the students who took the questionnaire, 70.1% have CVS. Increasing CVS severity was correlated with the number of hours of online learning and total hours of digital device usage (P<0.001). In contrast, the age of students is inversely correlated with the severity of CVS (P<0.001).(Figure 1)

The most common symptoms from CVS-Q were, headaches (n=1921, 77.58%), burning (n=1791, 72.33%) and eye pain (n=1767, 71.37%), respectively. In terms of severity, headaches were the most severe symptom (n=392, 15.83%) followed by worsening of eyesight (n=266, 10.74%), and pain (n=256, 10.34%).(Figure 2)

Risk Factors

Univariate analysis revealed multiple risk factors associated with CVS including, female gender (P ≤0.001), device usage without additional protection (blue-coated glasses, blue-coated film, or both) (P=0.006). Students who do their online studying with fans have higher risk of developing CVS, in comparison with students who study in an air-conditioned environment (P<0.001). Students who prefer using laptop computers have the greatest risk of developing CVS (OR=2.36, 95%CI: 1.75-3.19, P<0.001). (Table 3)

Multivariate analysis showed that significant risk factors of CVS were, age ≤ 15 years (AOR=2.17, 95%CI: 1.36-3.45, P=0.01), digital device usage > 6 hours/day (AOR=1.91 , 95%CI: 1.13-3.23, P=0.016), online learning > 5 hours/day (AOR=4.99, 95%CI: 3.08-8.12, P<0.001), multiple digital device usage for online learning (AOR=2.15, 95%CI: 1.04-4.43, P=0.038), refractive errors (AOR=2.89, 95%CI: 1.83-4.54, P<0.001), presence of back pain (AOR=2.06, 95%CI: 1.32-3.22, P=0.001), and presence of neck pain (AOR=2.36, 95%CI: 1.89-3.70, P<0.001). Myopia and emmetropia were independent risk factors of CVS (AOR=2.11, 95%CI: 1.24-3.32 and AOR=2.09, 95%CI: 2.14-3.47 respectively, P<0.001).

Discussion

Confidential: For Review Only

During the COVID-19 pandemic, the lockdown strategy was used to control the spread of the disease so schools were closed and online learning replaced the normal classroom environment. Digital device usage during this period increased over 4 hours per day, with the average of online learning at 7 hours per day. The prevalence of CVS in Thai students was 70.1%. CVS severity correlated with the number of hours for digital device usage and the number hours of online learning, while the age of student was inversely correlated CVS severity. The most common symptoms of CVS were, headaches (77.58%), burning (72.33%), and eye pain (71.37%), respectively. Multiple digital device usage during online learning, refractive error, the presence of neck and back pain, are also independent risk factors for CVS.

Each country has adopted the use of digital devices differently, whether for education, work, or leisure. Before the COVID-19 era, the prevalence of CVS among university students, adults, and office workers was between 60-80%. A study found that the prevalence of CVS was higher among engineering students compared to medical students. (13) Eyestrain was found in 18% of teenage students at the end of the day after working on digital devices. (14)

Increased online learning was reported in all levels of education. (15, 16) Students previously used digital devices as homework aids and for reading textbooks. Since school closures, digital devices were used for online classes as well, adding to the number of hours of screen time per day. The increased number of hours spent on digital devices were mostly for educational purposes.(17) During virtual learning of the COVID-19 pandemic, 50% of students in India had CVS, while 77% of students in China reported having at least one symptom of CVS. (17-19)

Among those with CVS, the severity was significantly correlated with the number of hours of screen time and age. Use of electronic devices over 5 hours was found to be associated with symptoms of CVS. (20) The use of mobile touch screen devices was related to the development of musculoskeletal symptoms. (21)The most common symptom of CVS was headache, in line with many published results. (19, 20, 22)

Students under 15 were twice more likely to have CVS than those over 15 years, with higher severity in younger students. Prevalence of CVS was lower in older aged Spanish university students than the younger group. (22) In contrast, China found that CVS was independently associated with older age.(18) These difference may be attributed to

Confidential: For Review Only

differences in the mean age group of students who have varied use of digital devices. Number of hours of digital device usage varied across grades. (23) Another possible explanation for different correlations with age may be the differences in age groups. Younger students might have problems answering some parts of the questionnaire. A study found that children and adolescents aged 6-17 years were unable to report symptoms of dry eye correctly. (24)

Multiple digital device usage is another independent risk factor for the development of CVS. Each type of device has it's own viewing distance. Reading and writing distance is usually 30-40 cm from the eyes. Lesser eyestrain was found when the computer monitor is 50-70 cm from the eyes. (25) As for mobile phones and tablets, which have smaller screens than computers, are usually held closer at about 20-30 cm from the eyes. (26) Half of the students in this study reported their reading distance between 40-80 cm, which is the appropriate distance for computers. Only 30% and 38.9% of students use laptop and desktop computers, respectively. About half of the students use multiple devices. When alternating between devices, students might not adjust viewing distance accordingly, leading to CVS. Laptop computers, tablets, and smartphones, are typically held in downward gaze. Increased corneal exposure from higher gaze angle results in increased tear evaporation. Variations in gaze position when alternating between devices lead to CVS. (27)

Like viewing distance, different types of digital devices require different posturing. Improper ergonomics lead to neck and back pain. Neck pain and back pain are independent risk factors which correlate with earlier reports. (20, 28, 29) A study found that pattern of smartphone or tablet usage in bouts of one hour or more was a risk for musculoskeletal symptoms, rather than total duration of use in number of hours per day. (21) Some students have reported lying on the bed while studying. (14)

Having refractive error adds to the risk of developing CVS, particularly myopia. Similar results from China found that self-reported myopic students who did and did not wear glasses had higher risk of CVS compared to children who were not myopic. (18) Children with myopia could possibly have residual near-work induced transient myopia from impaired sympathetic function, eventually leading to permanent myopic progression. (30) Long hours of online studying might lead to myopic progression causing children to wear under-corrected lenses. Residual refractive errors added with continued near-work studying can lead to CVS.

Confidential: For Review Only

Myopic and hyperopic students in our study were those who reported wearing myopic or hyperopic corrective lenses for their refractive errors. Those classified as emmetropic (28.8%) were those who did not use corrective lenses because our questionnaire asked whether students wore glasses or contact lenses for short- or long-sightedness, not whether they have refractive errors. Students with uncorrected refractive errors could be hidden in this group, which may be the reason why emmetropia was a significant risk factor for developing CVS. Patients with uncorrected refractive errors were at a higher risk than those with corrected refractive errors and those without refractive errors.

This study provides comprehensive data on CVS during the COVID-19 pandemic from high school students using a validated questionnaire. Not only students, but also parents, teachers, and schools could benefit from this study in terms of developing appropriate guidelines for online learning. Learning schedules should be adjusted to have appropriate durations and breaks. The overall hour of digital device usage should be under 6 hours/day and online learning should be limited to 5 hours/day, especially in younger students. This study emphasizes the need for regular eye examinations for students especially those with refractive errors, which should be fully corrected. Proper ergonomics and learning environment is also important. Students should adjust posturing and viewing distance according to the digital device used.

The main limitation of our study is that it is self-reported, which is subjective and some parameters such as refractive errors were not evaluated by standard methods. Second, students in this study were students in Bangkok, which does not represent students in Thailand. Each city in Thailand has been affected differently by the COVID pandemic, and each school has their own protocol of teaching. Lastly, we did not have students perform the CVS-Q before the pandemic, therefore we do not have data for comparison of CVS before and during lockdown.

Conclusion

During the pandemic, students spend an increasing number of hours on digital devices. Over 70% of students have CVS, with headache being the most frequent symptom. The number of hours on digital devices, the number of hours of online learning, refractive

Confidential: For Review Only

error, multiple digital device usage, presence of back pain, presence of neck pain, and younger age are contributory factors of developing CVS.

Online learning has grown over the years since before the COVID-19 pandemic, but has expanded exponentially during times of social distancing. We believe that even after this pandemic, online learning will remain, along with CVS. Our study points out factors associated with CVS, which we hope could be taken into consideration in remodeling our education system accordingly.

Acknowledgments

We would like to acknowledge Miss Benyapa Dechanupat, Mister Aphiphu Ruamthanthong, Miss Warisara Wattanasiripong, and Miss Alisha Kewkacha who helped distribute this survey.

Authors'contributions

Conceptualization: KS, TT; Data collection: KS, WS, NY; Methodology: KS, WT, NS, PS, TT; Statistical analysis: KS, NS, TT ; Writing-original draft: KS, WT, NS, PS, NY, TT; Writing-review&editing: KS, WT, WS, NS, PS, NY, TT

Funding statement

This research received no specific grant from any funding agency in the public, commercial or not-for-profit sectors

A competing interests statement

Not applicable, there are no competing interest

Patient and public involvement

Patient and/or the public were not involved in the design, or conduct, or reporting, or dissemination of this research

Patient consent form

Not applicable

Confidential: For Review Only

Ethics approval

This study has been approveds by the Ethics Committee of Rajavithi Hospital approved the protocol (Number 64237)

Data availability statement

The dataset used and/or analyzed during the current study are available from the corresponding author on reasonable request

Reference

1. Liu YC, Kuo RL, Shih SR. COVID-19: The first documented coronavirus pandemic in history. Biomedical journal. 2020;43(4):328-33.

2. Huang C, Wang Y, Li X, Ren L, Zhao J, Hu Y, et al. Clinical features of patients infected with 2019 novel coronavirus in Wuhan, China. Lancet (London, England). 2020;395(10223):497-506.

3. Xiao X, Newman C, Buesching CD, Macdonald DW, Zhou Z-M. Animal sales from Wuhan wet markets immediately prior to the COVID-19 pandemic. Scientific reports. 2021;11(1):11898.

4. World Health Organization. WHO Director-General's opening remarks at the media briefing on COVID-19-11 March 2020. Available at: https://www.who.int/director general/speeches/detail/who-director-general-s-opening-remarks-at-the-media-briefing-on-covid-19--11-march-2020. Accessed December 29, 2021.

5. Ferguson N, Laydon D, Nedjati-Gilani G, Imai N, Ainslie K, Baguelin M, et al. Report 9: Impact of non-pharmaceutical interventions (NPIs) to reduce COVID-19 mortality and healthcare demand2020.

Confidential: For Review Only

6. Eubank S, Eckstrand I, Lewis B, Venkatramanan S, Marathe M, Barrett CL. Commentary on Ferguson, et al., "Impact of Non-pharmaceutical Interventions (NPIs) to Reduce COVID-19 Mortality and Healthcare Demand". Bulletin of mathematical biology. 2020;82(4):52.

7. United Nations Educational, Scientific and Cultural Organization Institue for statistics : School closures and regional policies to mitigate learning loss due to COVID-19: a focus on the Asia-Pacific. Montreal, Quebec, Canada 2021.

8. Mick D, Fournier S. Paradoxes of Technology: Consumer Cognizance, Emotions, and Coping Strategies. Journal of Consumer Research. 1998;25(2):123-43.

9. The Economic Times. COVID-19 Impact : Screen time up by 100% for children. Available at: https://economictimes.indiatimes.com/industry/services/education/covid-19-impact-screen-time-up-by-100-for-children/articleshow/76383951.cms?from=mdr. Accessed December 29, 2021.

10. Twenge JM, Campbell WK. Associations between screen time and lower psychological well-being among children and adolescents: Evidence from a population-based study. Preventive Medicine Reports. 2018;12:271-83.

11. Daum KM, Clore KA, Simms SS, Vesely JW, Wilczek DD, Spittle BM, et al. Productivity associated with visual status of computer users. Optometry (St Louis, Mo). 2004;75(1):33-47.

12. Seguí Mdel M, Cabrero-García J, Crespo A, Verdú J, Ronda E. A reliable and valid questionnaire was developed to measure computer vision syndrome at the workplace. Journal of clinical epidemiology. 2015;68(6):662-73.

Confidential: For Review Only

13. Logaraj M, Madhupriya V, Hegde S. Computer vision syndrome and associated factors among medical and engineering students in chennai. Annals of medical and health sciences research. 2014;4(2):179-85.

14. Ichhpujani P, Singh RB, Foulsham W, Thakur S, Lamba AS. Visual implications of digital device usage in school children: a cross-sectional study. BMC ophthalmology. 2019;19(1):76.

15. Parajuli BR, Koirala S, Bajracharya A. Computer vision syndrome: a rising problem during COVID-19 period amongst students and online workers. Journal of Patan Academy of Health Sciences. 2021;8(2):6-9.

16. Wang L, Wei X, Deng Y. Computer Vision Syndrome During SARS-CoV-2 Outbreak in University Students: A Comparison Between Online Courses and Classroom Lectures. Frontiers in public health. 2021;9:696036.

17. Ganne P, Najeeb S, Chaitanya G, Sharma A, Krishnappa NC. Digital Eye Strain Epidemic amid COVID-19 Pandemic - A Cross-sectional Survey. Ophthalmic epidemiology. 2021;28(4):285-92.

18. Li R, Ying B, Qian Y, Chen D, Li X, Zhu H, et al. Prevalence of Self-Reported Symptoms of Computer Vision Syndrome and Associated Risk Factors among School Students in China during the COVID-19 Pandemic. Ophthalmic epidemiology. 2021:1-11.

19. Mohan A, Sen P, Shah C, Jain E, Jain S. Prevalence and risk factor assessment of digital eye strain among children using online e-learning during the COVID-19 pandemic: Digital eye strain among kids (DESK study-1). Indian journal of ophthalmology. 2021;69(1):140-4.

Confidential: For Review Only

20. Al Tawil L, Aldokhayel S, Zeitouni L, Qadoumi T, Hussein S, Ahamed SS. Prevalence of self-reported computer vision syndrome symptoms and its associated factors among university students. European journal of ophthalmology. 2020;30(1):189-95.

21. Toh SH, Coenen P, Howie EK, Smith AJ, Mukherjee S, Mackey DA, et al. A prospective longitudinal study of mobile touch screen device use and musculoskeletal symptoms and visual health in adolescents. Applied ergonomics. 2020;85:103028.

22. Cantó-Sancho N, Sánchez-Brau M, Ivorra-Soler B, Seguí-Crespo M. Computer vision syndrome prevalence according to individual and video display terminal exposure characteristics in Spanish university students. International journal of clinical practice. 2021;75(3):e13681.

23. Straker L, Harris C, Joosten J, Howie EK. Mobile technology dominates school children's IT use in an advantaged school community and is associated with musculoskeletal and visual symptoms. Ergonomics. 2018;61(5):658-69.

24. Hu L, Yan Z, Ye T, Lu F, Xu P, Chen H. Differences in children and adolescents' ability of reporting two CVS-related visual problems. Ergonomics. 2013;56(10):1546-57.

25. Rempel D, Willms K, Anshel J, Jaschinski W, Sheedy J. The effects of visual display distance on eye accommodation, head posture, and vision and neck symptoms. Human factors. 2007;49(5):830-8.

26. Long J, Cheung R, Duong S, Paynter R, Asper L. Viewing distance and eyestrain symptoms with prolonged viewing of smartphones. Clinical & experimental optometry. 2017;100(2):133-7.

27. Rosenfield M. Computer vision syndrome: a review of ocular causes and potential treatments. Ophthalmic & physiological optics : the journal of the British College of Ophthalmic Opticians (Optometrists). 2011;31(5):502-15.

Confidential: For Review Only

28. Teo C, Giffard P, Johnston V, Treleaven J. Computer vision symptoms in people with and without neck pain. Applied ergonomics. 2019;80:50-6.

29. Sen A, Richardson S. A study of computer-related upper limb discomfort and computer vision syndrome. Journal of human ergology. 2007;36(2):45-50.

30. Vasudevan B, Ciuffreda KJ, Gilmartin B. Sympathetic Inhibition of Accommodation after Sustained Nearwork in Subjects with Myopia and Emmetropia. Investigative Ophthalmology & Visual Science. 2009;50(1):114-20.

Confidential: For Review Only

Table 1 Demographic characteristics of participants and usage of electronic device before pandemic (N=2,476)

Demographic characteristics	N (%)
Mean age (years) ± SD	15.52 ± 1.66 (range 10-19)
Female	1,606 (64.9%)
**Grade**	
Grade 4-6	11 (0.4%)
Grade 7-9	781 (31.6%)
Grade 10-12	1684 (68%)
**Refractive status**	
Emmetropia	1180 (47.7%)
Myopia	1055 (42.6%)
Hyperopia	241 (9.7%)
**Glasses and Contact lens use**	
None	1180 (47.7%)
Glasses for myopia	784 (31.7%)
Glasses for hyperopia	186 (7.5%)
CL for myopia	151 (6.1%)
CL for hyperopia	47 (1.9%)
Glasses and CL for myopia	120 (4.8%)
Glasses and CL for hyperopia	8 (0.3%)
**Duration of digital device usage (pre-covid)**	
≤ 2h	201 (8.1%)
3-4 h	596 (24.1%)
5-6 h	648 (26.2%)
7-8 h	492 (19.9%)
9-10 h	401 (16.2%)
11-12 h	49 (1.9%)
> 12 h	89 (3.6%)
**Most digital device usage (pre-covid)**	
Mobile phone	1341 (54.2%)
Tablet	454 (18.3%)
Computer desktop	436 (17.6%)
Computer Laptop	216 (8.7%)
Television	29 (1.2%)
**Frequency of eye check-up**	
None	1054 (42.6%)
Once every 2 years	504 (20.4%)
Once a year	715 (28.9%)
Twice a year	203 (8.2%)

Confidential: For Review Only

1 **Table 2** The usage of electronic device on online learning during covid-19 lockdown (N=2,476)

Demographic characteristics	N (%)
**Total hours of digital device**	
≤ 4 h	84 (3.4%)
5-8 h	465 (18.8%)
9-12 h	1311 (52.9%)
13-16 h	571 (23.1%)
> 16 h	45 (1.8%)
**Total hours of online learning**	
≤ 2h	67 (2.7%)
3-4 h	271 (11%)
5-6 h	502 (20.3%)
7-8 h	1109 (44.8%)
9-10 h	480 (19.4%)
> 10 h	47 (1.8%)
**Device used for online learning**	
Single device	1486 (60%)
Multiple device	990 (40%)
Mobile phone	1174 (47.4%)
Tablet	1095 (44.2%)
Computer desktop	965 (39%)
Computer Laptop	691 (27.9%)
Television	27 (1.1%)
**Environment**	
Fan	1272 (51.4%)
Air-condition	1203 (48.6%)
**Protective instrument**	
None	413 (16.7%)
Blue-coated glasses	1172 (47.3%)
Blue coated film on digital devices	680 (27.5%)
Both blue-coated glasses and film on digital device	211 (8.5%)
**Frequency of eye rest**	
Never	371 (15%)
Every 15 minutes	596 (24.1%)
Every 30 minutes	431 (17.4%)
Every 45 minutes	304 (12.3%)
Every 1 hours	427 (17.2%)
Every ≥ 1 hour	347 (14%)
**Distance of digital device from eye during online learning**	
< 40 cm	943 (38.1%)
40-80 cm	1260 (50.9%)
> 80 cm	273 (11%)
**Back pain**	1880 (75.9%)
**Neck pain**	1687 (68.1%)
**Activity when rest**	
Close your eye	1144 (46.2%)
Sleep	1230 (49.7%)
Look out	822 (33.2%)
Play games	568 (22.9%)
Artificial tear	271 (10.9%)

Confidential: For Review Only

Table 3 The multivariate logistic regression the associated risk factor and CVS

Factor	Univariate analysis			Multivariate analysis		
	Crude OR	95%CI	P-value	Adjusted OR	95%CI	P-value
Age ≤ 15	2.34	1.63-3.36	<0.001	2.17	1.36-3.45	0.010
Female	1.73	1.34-2.23	<0.001	1.26	0.90-1.75	0.178
Overall digital usage > 6 hr	7.41	5.52-9.96	<0.001	1.91	1.13-3.23	0.016
Online learning > 5 hr	7.99	6.07-10.53	<0.001	4.99	3.08-8.12	<0.001
Refractive error	1.35	1-02-1.79	0.035	2.89	1.83-4.54	<0.001
**Refractive error**						
Myopia	3.21	2.31-4.44	<0.001	2.11	1.24-3.32	<0.001
Emmetropia	3.19	2.21-4.60	<0.001	2.09	2.14-3.47	<0.001
Hyperopia	Ref			Ref		
Fan	2.55	1.86-3.48	<0.001	1.20	0.81-1.81	0.362
Non-Protective device used	1.82	1.19-2.79	0.006	1.07	0.66-1.73	0.793
**Protective device**						
Both	4.62	2.90-7.38	<0.001	0.66	0.39-1.11	0.118
Blue-coated glasses	3.36	2.52-4.49	<0.001	1.19	0.71-1.99	0.508
Blue-coated film	Ref			Ref		
Rest over 45 mins	2.59	2.01-3.34	<0.001	1.02	0.7-1.48	0.935
Other digital device used > 2 hr	2.22	1.73-2.85	<0.001	0.89	0.61-1.28	0.520
Distance from device < 40 cm	2.42	1.78-3.30	<0.001	1.07	0.71-1.63	0.743
Back pain	2.84	2.12-3.80	<0.001	2.06	1.32-3.22	0.001
Neck pain	2.59	1.94-3.46	<0.001	2.64	1.89-3.70	<0.001
Multiple digital device used	3.60	2.76-4.70	<0.001	2.15	1.04-4.43	0.038
Non Use Artificial tear	0.96	0.70-1.30	0.77	1.34	0.91-1.98	0.133
Close eye during online learning	1.69	1.32-2.17	<0.001	1.12	0.80-1.55	0.508
Sleep during online learning	2.12	1.64-2.74	<0.001	1.29	0.93-1.79	0.135
Laptop computers preference	2.36	1.75-3.19	<0.001	0.83	0.48-1.42	0.494
Tablet preference	2.27	1.77-2.92	<0.001	0.86	0.51-1.45	0.568
Television screen preference	1.49	0.49-4.55	0.49	1.067	0.30-3.75	0.920
Computer desktop preference	1.37	1.07-1.77	0.016	1.00	0.61-1.66	0.987
Mobile phone preference	1.34	1.03-1.69	0.031	0.81	0.50-1.31	0.395

Confidential: For Review Only

Figure 1 Incidence and severity of CVS according the mean hour of online learning and the mean hour of digital device

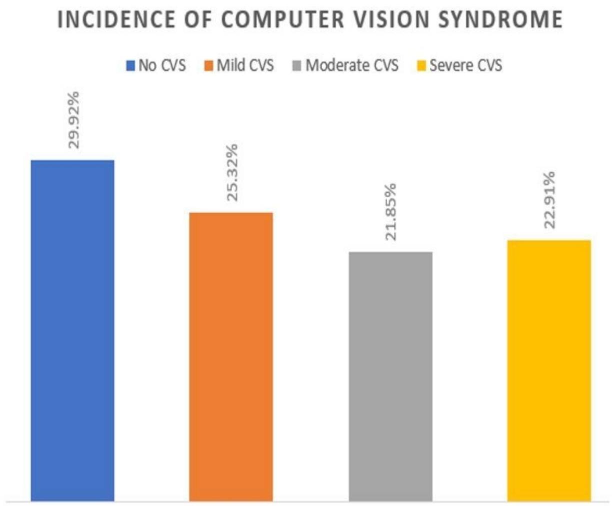

	No CVS (n=741)	Mild CVS (n=627)	Moderate CVS (n=541)	Severe CVS (n=567)	P-value
Mean age (yrs) ± SD	15.55 ± 1.11	15.35 ± 1.27	15.20 ± 1.22	15.10 ± 1.14	< 0.001
The mean hour of online learning (hrs) ± SD	6.16 ± 2.26	7.23 ± 2.01	7.28 ± 1.78	7.69 ± 1.74	< 0.001
The mean hour of total digital device (hrs) ± SD	9.45 ± 3.38	10.77 ± 2.93	10.89 ± 2.57	11.33 ± 2.54	< 0.001

101x87mm (300 x 300 DPI)

Figure 2 The percentage of frequency and severity of different eye symptoms of CVS in student

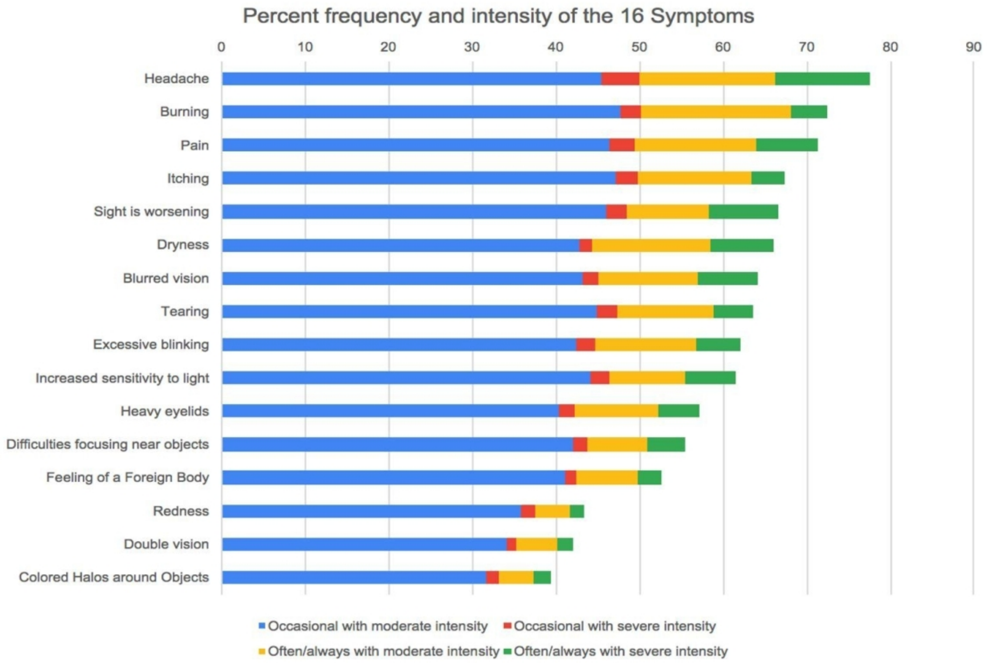

128x91mm (300 x 300 DPI)

Effects of digital devices and online learning on computer vision syndrome in students during the COVID-19 era

<u>Survey</u>

The purpose of this survey: This survey aims to gather information from students of different year groups about what visual symptoms they experience and how well they protect their eyes during the period of online learning, since it is compulsory for students to sit in front of their screens for a long period of time. This results of this survey will then be analyzed, conclusion will be drawn and the results will then be incorporated as one of the research findings.

General overview of the survey:
First section of the survey - 8 questions
Second section of the survey - 8 questions
Third section of the survey - 18-36 questions

It will take approximately 2 minutes to fill out the survey!

Please note that the survey will be anonymous. The information will be kept confidential and will only be used for this research.

Informed consent

1. Do you voluntarily agree to complete this survey?
    - ○ I voluntarily agreed to complete this survey

Section 1 of the survey: General information

1. Age

    ____

2. Gender
    - ○ Male
    - ○ Female
3. Grade level
    - ○ Grade 1-3/Year 2-4
    - ○ Grade 4-6/Year 5-7
    - ○ Grade 7-9/Year 8-10
    - ○ Grade 10-12/Year 11-13
4. Before the period of online learning, how many hours per day do you spend on average using electronic devices?
    - ○ 0 hour
    - ○ 1 hour
    - ○ 2 hours
    - ○ 3 hours
    - ○ 4 hours
    - ○ 5 hours
    - ○ 6 hours
    - ○ 7 hours

Confidential: For Review Only

- 8 hours
- 9 hours
- 10 hours
- 11 hours
- 12 hours
- Other……………..

5. Before the period of online learning, what electronic devices did you use most often?
   - Tablet (iPad)
   - Mobile Phone
   - Computer/Desktop
   - Laptop
   - Television
   - Other
6. Do you wear any of these? (Can tick more than one box)
   - Glasses for short-sightedness
   - Glasses for long-sightedness
   - Contact lenses for short-sightedness
   - Contact lenses for long-sightedness

7. How often do you have an eye check-up?
   - Never
   - Once every two years
   - Once a year
   - Twice a year
   - Other

Section 2 of the Survey

7. What electronic devices do you use for online learning?
   - Tablet (iPad)
   - Mobile Phone
   - Computer/Desktop
   - Laptop
   - Television
   - Other
8. How many hours per day do you spent learning online?
   - 1 hour
   - 2 hours
   - 3 hours
   - 4 hours
   - 5 hours
   - 6 hours
   - 7 hours
   - 8 hours
   - 9 hours
   - 10 hours
   - 11 hours

Confidential: For Review Only

- 12 hours
- Other……………..

9. What is the environment of your room when you learn online? Do you use a fan or an air conditioner?
    - Fan
    - Air conditioner
    - Other
10. Do you use any equipment to help protect your eyes?
    - Glasses
    - Films for computer/tablets/phone screens
11. How often do you give your eyes a rest during online classes?
    - Never
    - Every 15 minutes
    - Every 30 minutes
    - Every 45 minutes
    - Every 1 hour
    - Every 2 hours
    - Other
12. What do you do when you rest your eyes?
    - Close your eyes
    - Look out of the window as far as you could
    - Play games on your device
    - Sleep
    - Artificial tear
    - Other
13. Apart from using electronic devices for online learning, how much time do you spend using electronic devices for other activities?
    - 1 hour
    - 2 hours
    - 3 hours
    - 4 hours
    - 5 hours
    - 6 hours
    - 7 hours
    - 8 hours
    - 9 hours
    - 10 hours
    - 11 hours
    - 12 hours
    - Other……………..
14. How far apart do you position your devices from your eyes?
    - 20-40 cm
    - 40-60 cm
    - 60-80 cm
    - 80-100 cm
    - more than 100 cm

Confidential: For Review Only

Section 3 of the survey:
This section will be the computer vision syndrome questionnaire.

The first question will be related to the frequency of the symptom, how often the symptom occurs:
Never = the symptom does not occur at all
Occasionally = Sporadic episodes or once a week
Often or always = 2 or 3 times a week or almost everyday

NOTE: If you have answered "Never" for the first question, you will not be asked about the intensity, but if you answered "Occasionally" or "Often or always", you will be required to answer the question about intensity.

The second question will be related to the intensity of the symptom. You are required to choose between "moderate" or "Intense" for each of the symptoms.

15. When you learn online, do you experience the symptom of eye burning?
    - Never (the symptom does not occur)
    - Occasionally (once a week)
    - Often or always (2 or 3 times a week or almost everyday)
16. What is the intensity of the symptom? (If you answered never, do not answer this question)
    - Moderate
    - Intense
17. When you learn online, do you experience the symptom of eye itching?
    - Never (the symptom does not occur)
    - Occasionally (once a week)
    - Often or always (2 or 3 times a week or almost everyday)
18. What is the intensity of the symptom? (If you answered never, do not answer this question)
    - Moderate
    - Intense
19. When you learn online, have you ever feel like there's foreign body in your eyes?
    - Never (the symptom does not occur)
    - Occasionally (once a week)
    - Often or always (2 or 3 times a week or almost everyday)
20. What is the intensity of the symptom? (If you answered never, do not answer this question)
    - Moderate
    - Intense
21. When you learn online, do you experience the symptom of tearing?
    - Never (the symptom does not occur)
    - Occasionally (once a week)
    - Often or always (2 or 3 times a week or almost everyday)
22. What is the intensity of the symptom? (If you answered never, do not answer this question)
    - Moderate

Confidential: For Review Only

- Intense

23. When you learn online, do you experience the symptom of excessive blinking?
    - Never (the symptom does not occur)
    - Occasionally (once a week)
    - Often or always (2 or 3 times a week or almost everyday)
24. What is the intensity of the symptom? (If you answered never, do not answer this question)
    - Moderate
    - Intense
25. When you learn online, do you experience the symptom of eye redness?
    - Never (the symptom does not occur)
    - Occasionally (once a week)
    - Often or always (2 or 3 times a week or almost everyday)
26. What is the intensity of the symptom? (If you answered never, do not answer this question)
    - Moderate
    - Intense
27. When you learn online, do you experience the symptom of eye pain?
    - Never (the symptom does not occur)
    - Occasionally (once a week)
    - Often or always (2 or 3 times a week or almost everyday)
28. What is the intensity of the symptom? (If you answered never, do not answer this question)
    - Moderate
    - Intense
29. When you learn online, do you experience the symptom of heavy eyelids?
    - Never (the symptom does not occur)
    - Occasionally (once a week)
    - Often or always (2 or 3 times a week or almost everyday)
30. What is the intensity of the symptom? (If you answered never, do not answer this question)
    - Moderate
    - Intense
31. When you learn online, do you experience the symptom of eye dryness?
    - Never (the symptom does not occur)
    - Occasionally (once a week)
    - Often or always (2 or 3 times a week or almost everyday)
32. What is the intensity of the symptom? (If you answered never, do not answer this question)
    - Moderate
    - Intense
33. When you learn online, do you experience the symptom of blurred vision?
    - Never (the symptom does not occur)
    - Occasionally (once a week)
    - Often or always (2 or 3 times a week or almost everyday)
34. What is the intensity of the symptom? (If you answered never, do not answer this question)

Confidential: For Review Only

- Moderate
- Intense

35. When you learn online, do you experience the symptom of double vision?
    - Never (the symptom does not occur)
    - Occasionally (once a week)
    - Often or always (2 or 3 times a week or almost everyday)
36. What is the intensity of the symptom? (If you answered never, do not answer this question)
    - Moderate
    - Intense
37. When you learn online, how often do you experience difficulties focusing near objects?
    - Never (the symptom does not occur)
    - Occasionally (once a week)
    - Often or always (2 or 3 times a week or almost everyday)
38. What is the intensity of the symptom? (If you answered never, do not answer this question)
    - Moderate
    - Intense
39. When you learn online, do you experience the symptom of increased sensitivity to light?
    - Never (the symptom does not occur)
    - Occasionally (once a week)
    - Often or always (2 or 3 times a week or almost everyday)
40. What is the intensity of the symptom? (If you answered never, do not answer this question)
    - Moderate
    - Intense
41. When you learn online, do you experience the symptom of colored halos around objects?
    - Never (the symptom does not occur)
    - Occasionally (once a week)
    - Often or always (2 or 3 times a week or almost everyday)
42. What is the intensity of the symptom? (If you answered never, do not answer this question)
    - Moderate
    - Intense
43. When you learn online, do you experience the feeling that sight is worsening?
    - Never (the symptom does not occur)
    - Occasionally (once a week)
    - Often or always (2 or 3 times a week or almost everyday)
44. What is the intensity of the symptom? (If you answered never, do not answer this question)
    - Moderate
    - Intense
45. When you learn online, how often do you have a headache?
    - Never (the symptom does not occur)

Confidential: For Review Only

- Occasionally (once a week)
- Often or always (2 or 3 times a week or almost everyday)

46. What is the intensity of the symptom? (If you answered never, do not answer this question)
    - Moderate
    - Intense
47. When you learn online, how often do you experience back pain?
    - Never (the symptom does not occur)
    - Occasionally (once a week)
    - Often or always (2 or 3 times a week or almost everyday)
48. What is the intensity of the symptom? (If you answered never, do not answer this question)
    - Moderate
    - Intense
49. When you learn online, how often do you experience neck pain?
    - Never (the symptom does not occur)
    - Occasionally (once a week)
    - Often or always (2 or 3 times a week or almost everyday)
50. What is the intensity of the symptom? (If you answered never, do not answer this question)
    - Never (the symptom does not occur)
    - Occasionally (once a week)
    - Often or always (2 or 3 times a week or almost everyday)

Thank you for completing this survey!

Confidential: For Review Only

BMJ Paediatrics Open

Effects of digital devices and online learning on computer vision syndrome in students during the COVID-19 era: an online questionnaire study

Journal:	*BMJ Paediatrics Open*
Manuscript ID	bmjpo-2022-001429.R1
Article Type:	Original research
Date Submitted by the Author:	09-Apr-2022

Complete List of Authors:	Seresirikachorn, Kasem; Thammasat University Sirindhorn International Institute of Technology<br>Thiamthat, Warakorn; Rajavithi Hospital, Department of Ophthalmology, College of Medicine, Rangsit University<br>Sriyuttagrai, Wararee; Rajavithi Hospital, Department of Ophthalmology, College of Medicine, Rangsit University<br>Soonthornworasiri, Ngamphol; Mahidol University, Department of Tropical Hygiene<br>Singhanetr, Panisa; Metta Pracharak Hospital Wat Rai Khing, Department of Ophthalmology<br>Yudtanahiran, Narata; Chulalongkorn University Faculty of Medicine<br>Theeramunkong, Thanaruk; Thammasat University Sirindhorn International Institute of Technology; Royal Society of Thailand

Keywords:	COVID-19, Adolescent Health, Information Technology, Ophthalmology, Epidemiology

SCHOLARONE™
Manuscripts

I, the Submitting Author has the right to grant and does grant on behalf of all authors of the Work (as defined in the below author licence), an exclusive licence and/or a non-exclusive licence for contributions from authors who are: i) UK Crown employees; ii) where BMJ has agreed a CC-BY licence shall apply, and/or iii) in accordance with the terms applicable for US Federal Government officers or employees acting as part of their official duties; on a worldwide, perpetual, irrevocable, royalty-free basis to BMJ Publishing Group Ltd ("BMJ") its licensees and where the relevant Journal is co-owned by BMJ to the co-owners of the Journal, to publish the Work in this journal and any other BMJ products and to exploit all rights, as set out in our licence.

The Submitting Author accepts and understands that any supply made under these terms is made by BMJ to the Submitting Author unless you are acting as an employee on behalf of your employer or a postgraduate student of an affiliated institution which is paying any applicable article publishing charge ("APC") for Open Access articles. Where the Submitting Author wishes to make the Work available on an Open Access basis (and intends to pay the relevant APC), the terms of reuse of such Open Access shall be governed by a Creative Commons licence – details of these licences and which Creative Commons licence will apply to this Work are set out in our licence referred to above.

Other than as permitted in any relevant BMJ Author's Self Archiving Policies, I confirm this Work has not been accepted for publication elsewhere, is not being considered for publication elsewhere and does not duplicate material already published. I confirm all authors consent to publication of this Work and authorise the granting of this licence.

Confidential: For Review Only

Effects of digital devices and online learning on computer vision syndrome in students during the COVID-19 era: an online questionnaire study

Kasem Seresirikachorn, MD [1]
Warakorn Thiamthat, MD [2]
Wararee Sriyuttagrai, MD [2]
Ngamphol Soonthornworasiri, PhD [3]
Panisa Singhanetr, MD [4]
Narata Yudtanahiran [5]
Thanaruk Theeramunkong, PhD [1,6]

[1] Sirindhorn International Institute of Technology, Thammasat University, Pathumthani, Thailand.
[2] Department of Ophthalmology, College of Medicine, Rangsit University, Rajavithi Hospital, Bangkok, Thailand.
[3] Department of Tropical Hygiene, Faculty of Tropical Medicine, Mahidol University, Bangkok, Thailand.
[4] Department of Ophthalmology, Mettapracharak Hospital, Nakhon Pathom, Thailand.
[5] Faculty of Medicine, Chulalongkorn University, Bangkok, Thailand.
[6] The Royal Society of Thailand, Bangkok, Thailand.

Running title: Effects of digital devices and online learning on computer vision syndrome in students during the COVID-19 era: an online questionnaire study

None of the authors have any conflict of interest to disclose

Correspondence: Thanaruk Theeramunkong PhD,
Sirindhorn International Institute of Technology, Thammasat University,
131 Moo5, Tiwanont Road,
Bangkadi, Muang
Pathumthani 12000, Thailand.
Tel: 66 2-501-3505 ext 6018
Fax: 66 2-501-3505 ext 6014
Email: thanaruk@siit.tu.ac.th

Confidential: For Review Only

Effects of digital devices and online learning on computer vision syndrome in students during the COVID-19 era: an online questionnaire study

Abstract (280 words)

Purpose : Computer vision syndrome (CVS) describes a group of eye and vision-related problems that result from prolonged digital device use. This study aims to assess the prevalence and associated factors of CVS among students during the lockdown resulting from the COVID-19 pandemic.

Methods: A cross-sectional, online, questionnaire-based study performed among high school students in Thailand.

Results: A total of 2,476 students, with mean age of 15.52 ± 1.66 years, were included in this study. The mean number of hours of digital device use per day (10.53 ± 2.99) increased during the COVID-19 pandemic compared to before its advent (6.13 ± 2.8). The mean number of hours of online learning was 7.03 ± 2.06 hours per day during the pandemic. CVS was found in 70.1% of students, and its severity correlated with both the number of hours of online learning and the total number of hours of digital device usage ($P<0.001$). Multiple logistic regression analysis revealed that the factors associated with CVS included age ≤ 15 years (adjusted odds ratio (AOR)=2.17), overall digital device usage > 6 hours per day (AOR=1.91), online learning > 5 hours per day (AOR=4.99), multiple digital device usage (AOR=2.15), refractive errors (AOR=2.89), presence of back pain (AOR=2.06), and presence of neck pain (AOR=2.36).

Conclusions: The number of hours of digital device usage increased during lockdown. Over 70% of children had CVS, whose associated factors, including hours of digital device usage, hours of online learning, ergonomics, and refractive errors, should be adjusted to decrease the risk of acquiring this condition. Online learning will remain, along with CVS, after this pandemic, and we hope our research will be taken into account in remodeling our education system accordingly.

Keywords: Coronavirus disease (COVID-19), computer vision syndrome (CVS), digital eye strain, severe acute respiratory syndrome coronavirus 2 (SARS-CoV-2)

Confidential: For Review Only

Key Messages

What is known about the subject

- Computer vision syndrome describes a group of vision-related problems caused by prolonged use of digital devices.
- Learning has shifted from being performed in schools to taking place online during the COVID-19 pandemic.

What this study adds

- Digital device use has increased by 4 hours per day, and the mean hours of online learning was 7 hours per day.
- Computer vision syndrome was found in 70.1% of students.
- Factors associated with CVS included digital device usage > 6 hours/day, multiple digital device usage, refractive errors, and presence of back and neck pain.

1. Background

In 2019, there was a report from Wuhan Municipal Health Commission about a cluster of cases of pneumonia in Wuhan, China. (1-3) What started as an outbreak in China is now a global crisis. On March 11, 2020, the WHO announced that COVID-19 could be classified as a pandemic. (4) Suppression measures aimed at slowing down epidemic growth by reducing the number of cases and human to human transmission included social distancing and closure of schools and universities, and (5, 6) UNESCO reported that 1.37 billion students from over 130 countries were affected by these interventions. For Thailand, the total duration of school closure was 42 weeks. (7)

Many schools shifted from classroom-based learning to online schooling in order to continue teaching. Up to 54% of parents of children aged 5 to 15 years reported as many as 5 additional hours spent online. (8) Moderate use of screens (4 hours/day) was associated with lower psychological well-being, including less curiosity, lower self-control, distractibility, and inability to finish tasks. (9)

Rapid advancement in technology has led to digital devices becoming a big part of our daily lives, especially for students, who use digital devices as homework aids, for reading, and for leisure activities. Approximately 40% of school pupils have been found to spend less than 2 hours a day or 2-4 hours a day on digital device reading, while 14% spent

Confidential: For Review Only

4-6 hours and 3% spent over 6 hours each day using digital devices for reading. (10) Prolonged screen time can produce physical discomfort known as digital eye strain (DES), or computer vision syndrome (CVS). The American Optometric Association (AOA) defined CVS as a group of eye and vision-related problems that result from prolonged usage of digital devices which cause increased stress to near vision. (11) The diagnosis of CVS is subjective, with numerous questionnaires being developed to diagnose this syndrome, including the CVSS17 questionnaire, a 6-item visual fatigue scale by Benedetto, and the Computer Vision Syndrome Questionnaire (CVS-Q) by Segui et al. (12-14) The CVS-Q is a validated questionnaire commonly used in clinical trials to evaluate the visual health of digital device users.

Before the COVID-19 era, the prevalence of CVS among university students, adults, and office workers was between 60-80%. (15) Eyestrain was found in 18% of teenage students at the end of the day after working on digital devices. (10) A study from Indonesia reported that 87.2% of high school students experienced evaporative dry eye, which is one of the risk factors of CVS. (16) On average, children aged 8-12 years in the United States were found to spend 4-6 hours a day watching or using screens, while teens spent up to 9 hours.(17) In Thailand, 94.84% of secondary school students were found to have at least one symptom of CVS in 2016. Thai children spend approximately 35 hours per week watching screens. (18)

The prevalence of dry eye symptoms is greater during electronic screen use than when viewing printed materials. (19) Environmental factors such as use of air conditioning and windy environments have been reported to correlate with visual symptoms of dry eye disease, (20) which is a major contributor to CVS. (21) Factors previously reported to be associated with CVS were hours of use, screen distance, screen brightness, room illumination, wearing of contact lenses, and refractive errors. (10, 22) Commonly-reported symptoms relating to CVS are headaches, eyestrain, blurred vision, dry eye symptoms, and pain in the neck and shoulders.

This study aims to assess the prevalence and associated factors of CVS among school students in Bangkok during lockdown resulting from the COVID-19 pandemic.

2. Materials and Methods

This cross-sectional, online, questionnaire-based study was approved by the institutional review boards of Rajavithi Hospital, Thailand, and it was conducted in accordance with the tenets of the Declaration of Helsinki. An online questionnaire was sent to primary and secondary school students (ages 10-19 years) electronically. Before answering the questionnaire, all participants were informed on the cover letter of the questionnaire about the study's purpose, methods and guarantee of anonymity of data. All participants were required to sign and accept an informed consent form before continuing with the survey, in which they answered the questions themselves. Data was collected between August 16, 2021 and August 31, 2021 (15 days), during online schooling in accordance with the COVID-19 lockdown policy.

Online questionnaire

The questionnaire consisted of 4 parts: demographic data and electronic device usage before the pandemic; online learning behavior; a Computer Vision Syndrome Questionnaire (CVS-Q); and a poster providing students with information on the proper use of electronic devices. (Supplementary 1)

The CVS-Q, which was developed by Segui et al., (14) investigates the presence of 16 eye symptoms. High school students were required to report the frequency and intensity of each eye symptom. For each symptom, the frequency score is multiplied by the intensity score and all of the scores for each symptom are added together. For the person to be considered as having computer vision syndrome, he or she must have a total score of greater than or equal to six. The severity was divided into mild, moderate, and severe, corresponding with scores of 6-12, 13-18, and 19 or over respectively.

Statistical Analysis

All the data from the electronic survey was analyzed with SPSS 16.0 for Windows (SPSS Inc., Chicago, IL, USA). Frequencies and percentages were used for categorical data. Continuous data were reported using mean, median, and standard deviation (SD) after confirmation of normal distribution of the data. Paired-T test was used to compare the number of hours of digital device use during and prior to the COVID pandemic, while one-way

Confidential: For Review Only

ANOVA was utilized to compare hours of online learning, total hours of digital device usage, and severity of CVS. Risk factors associated with CVS were analyzed by univariate and multiple logistic regression to identify independent risk factors of CVS by calculating the odds ratio (OR) and their corresponding 95% confidence interval (CI). All variables with a *p*-value < 0.05 in the univariate analysis were further analyzed by multiple logistic regression. A *p*-value < 0.05 was considered statistically significant.

3. Results

<u>Baseline Characteristics</u>

A total of 2,476 students completed the online survey. Demographic data and electronic device usage before the pandemic is shown in Table 1. The participants' mean age was 15.52 ± 1.66 years with a female predominance (64.9%). The majority of students were in grades 10-12 (68%), and around 40% used glasses or contact lenses to correct their refractive errors. Mobile phones were the most used digital device before the COVID-19 pandemic at 54.2%, and a quarter of the students had around 5-6 hours of screen time before the lockdown. (Table 2)

<u>Digital Device Usage During the COVID-19 pandemic</u>

The mean number of hours spent per day using digital devices before and after the pandemic were 6.13 ± 2.8 and 10.53 ± 2.99, respectively (p< 0.001). (Table 2) Over 60% of the students spend at least 7 hours/day with online learning (mean = 7.03 ± 2.06 hours/day), and 40% of them used multiple devices when learning online, with mobile phones being the most used. The majority of students did their online studying in a fan-ventilated environment, and over 80% employed some sort of protective equipment, including blue-coated glasses or protective films on their digital devices. Symptoms of back pain and neck pain were present in around 75.9% and 68.1% of students respectively during lockdown. (Table 3)

<u>Computer Vision Syndrome Questionnaire</u>

The median CVS-Q score was 11 (range 0-64), and 70.1% of participants had CVS. Increasing severity of the condition was correlated with the number of hours of online learning and total hours of digital device usage (P<0.001). (Figure 1)

Confidential: For Review Only

The most common symptoms from CVS-Q were headaches (n=1921, 77.58%), burning (n=1791, 72.33%) and eye pain (n=1767, 71.37%). In terms of severity, headaches were the most severe symptom (n=392, 15.83%) followed by worsening of eyesight (n=266, 10.74%), and pain (n=256, 10.34%). (Figure 2)

Associated Factors

Univariate analysis revealed multiple factors associated with CVS, including female gender (P ≤0.001) and device usage without additional protection (blue-coated glasses, blue-coated film, or both) (P=0.006). Students who did their online studying with fans had a higher risk of developing CVS in comparison with students who studied in an air-conditioned environment (P<0.001), and those who preferred using laptop computers had the greatest risk of developing CVS (OR=2.36, 95%CI: 1.75-3.19, P<0.001). (Table 3)

Multiple logistic regression analysis showed that the significant factors associated with CVS were age ≤ 15 years (AOR=2.17, 95%CI: 1.36-3.45, P=0.01), digital device usage > 6 hours/day (AOR=1.91 , 95%CI: 1.13-3.23, P=0.016), online learning > 5 hours/day (AOR=4.99, 95%CI: 3.08-8.12, P<0.001), multiple digital device usage for online learning (AOR=2.15, 95%CI: 1.04-4.43, P=0.038), refractive errors (AOR=2.89, 95%CI: 1.83-4.54, P<0.001), presence of back pain (AOR=2.06, 95%CI: 1.32-3.22, P=0.001), and presence of neck pain (AOR=2.36, 95%CI: 1.89-3.70, P<0.001). Myopia and emmetropia were independent risk factors (AOR=2.11, 95%CI: 1.24-3.32 and AOR=2.09, 95%CI: 2.14-3.47 respectively, P<0.001).

4. Discussion

During the COVID-19 pandemic, a lockdown strategy was used to control the spread of the disease, so schools were closed and online learning replaced the normal classroom environment. Digital device usage during this period increased by over 4 hours per day, with a mean of 7 hours per day of online learning. The prevalence of CVS in Thai students was 70.1%, and its severity correlated with the number of hours of digital device usage and the number hours of online learning. The most common symptoms of CVS were headaches (77.58%), burning (72.33%), and eye pain (71.37%). Multiple digital device usage during online

Confidential: For Review Only

learning, refractive error, and the presence of neck and back pain, were also independent associated factors of CVS.

Increased online learning has been reported in all levels of education. (23, 24) Students previously used digital devices as homework aids and for reading textbooks, but after school closures, they were used for online classes as well, adding to the number of hours of screen time per day. The increase in hours spent on digital devices was mostly for educational purposes. (25) During virtual learning due to the COVID-19 pandemic, 50% of students in India had CVS, while 77% of students in China reported having at least one of its symptoms. (25-27)

Among those with CVS, the severity was significantly correlated with the number of hours of screen time, with the use of electronic devices for over 5 hours found to be associated with it. (22) The use of mobile touch screen devices was related to the development of musculoskeletal symptoms. (28)The most common symptom of CVS in our study was headaches, in line with the findings of many other published reports. (22, 27, 29)

Students under 15 were twice as likely to have CVS as their older counterparts, with the higher severity occurring in younger students. Prevalence of CVS was found to be lower in older Spanish university students than in the younger group. (29) In contrast, a Chinese study found that CVS was independently associated with older age.(26) These apparent anomalies may be attributed to variations in the mean age of students who have diverse levels of digital devices use. The number of hours of digital device usage varied across grades. (30) Another possible explanation for diverse correlations with age may be the developmental differences in age groups, as younger students might have had problems answering some parts of the questionnaire. A previous study found that children and adolescents aged 6-17 years were unable to report symptoms of dry eye correctly. (31)

Multiple digital device usage was another independent associated factor of CVS. Each type of device has its own recommended viewing distance. For computers, the reading and writing distance is usually 30-40 cm from the eyes, and less eyestrain was found when the computer monitor was 50-70 cm from the eyes. (32) Mobile phones and tablets, which have smaller screens, are usually held closer, at about 20-30 cm from the eyes. (33) Half of the students in this study reported their reading distance as between 40-80 cm, which is the

Confidential: For Review Only

appropriate distance for computers. Only 30% and 38.9% of students respectively used laptop and desktop computers, while about half used multiple devices. When alternating between devices, students may not adjust the viewing distance appropriately, and this could lead to symptoms of CVS. Laptop computers, tablets, and smartphones are typically held in downward gaze, and greater corneal exposure from higher gaze angles results in increased tear evaporation. Variations in gaze position when alternating between devices can lead to CVS. (19)

Just as different types of digital devices require different viewing positions, they also involve variations in posturing, and improper ergonomics can lead to neck and back pain, which were independent risk factors in our study, in agreement with the findings of earlier reports. (22, 34, 35) A previous study found that a pattern of using smartphones or tablets in bouts of one hour or more carried a higher risk of musculoskeletal symptoms than the total duration of use throughout the day. (28) Some students have reported lying on the bed while studying. (10)

Having refractive error, particularly myopia, adds to the risk of developing CVS. Similar results were found in research in China which concluded that self-reported myopic students, both who did and did not wear glasses, were at higher risk of CVS compared to those who were not myopic. (26) Children with myopia could possibly have residual near-work induced transient myopia from impaired sympathetic function, eventually leading to permanent myopic progression. (36) Long hours of online studying might lead to myopic progression, causing children to wear under-corrected lenses, and residual refractive errors combined with continued near-work studying has been found to lead to CVS.

Myopic and hyperopic students in our study were those who reported wearing myopic or hyperopic corrective lenses for their refractive errors; those classified as emmetropic (28.8%) reported not using corrective lenses because our questionnaire asked whether students wore glasses or contact lenses for short- or long-sightedness, not whether they had refractive errors. Around 40% of students reportedly never had eye examinations, while 20% receive one every 2 years. Students with some uncorrected refractive errors could be hidden in this group, which may be the reason why emmetropia was a significant risk factor for developing CVS. People with uncorrected refractive errors have been found to be at a higher risk than those with corrected refractive errors and those without refractive errors. (26)

Confidential: For Review Only

Prolonged exposure to computers in CVS patients has been reported to significantly correlate with dry eye disease.(37) Environmental factors producing corneal drying include low ambient humidity, high forced-air heating or air conditioning settings, together with the use of ventilation fans, excess static electricity, or airborne contaminants. (38) A study among information technology professionals in Egypt found that exposure to air pollution, use of air conditioners, and exposure to windy environments, were significant predictors of CVS. (39) Symptoms of dry eye are major components of CVS; (40) therefore, identifying environmental factors related to dry eye is necessary to make adjustments to minimize the condition.

This study provides comprehensive data on CVS during the COVID-19 pandemic from high school students using a validated questionnaire. Not only students, but also parents, teachers, and schools could benefit from using this study in terms of developing appropriate guidelines for online learning. Timetables should be adjusted to have appropriate durations and breaks. The overall digital device usage should be under 6 hours/day, and online learning should be limited to 5 hours/day, especially in younger students. This study emphasizes the need for regular eye examinations for students, especially those with refractive errors, which should be fully corrected. Proper ergonomics and learning environments are also important; students should adjust posturing and viewing distance according to the digital device used.

The main limitation of our study is that it used a self-reported questionnaire which is subject to bias. Due to the cross-sectional nature of this research, we were only able to identify factors associated with CVS, which has been defined by a mixture of symptoms and signs resulting in various definitions being used across clinical research, thereby limiting the available reports that could be compared with our results. The participants in this study were from a single city, so that it does not represent the online schooling situation in all of Thailand. Lastly, we did not have students perform the CVS-Q before the pandemic, so we do not have data for comparison of CVS before and during lockdown.

Further evaluations with objective methods such as tear break-up time, Schirmer test, and ocular surface staining could be added into the analysis. A possible pattern of online learning and its association with CVS could be investigated in a study of longer duration.

Confidential: For Review Only

5. Conclusion

During the pandemic, students have spent an increasing number of hours on digital devices, and over 70% have CVS, with headaches being the most frequent symptom. The number of hours spent on digital devices and online learning, refractive errors, multiple digital device usage, presence of back pain, presence of neck pain, and younger age are factors associated with CVS.

Online learning had already grown over the years before the COVID-19 pandemic, but it has expanded exponentially during times of social distancing. We believe that even after this pandemic, online learning will remain, along with CVS. Our study points out factors associated with this condition which we hope will be taken into consideration in remodeling our education system appropriately.

Acknowledgments

We would like to acknowledge the assistance of Miss Benyapa Dechanupat, Mr. Aphiphu Ruamthanthong, Miss Warisara Wattanasiripong, and Miss Alisha Kewkacha, who helped distribute this survey.

Authors' contributions

Conceptualization: KS, TT; Data collection: KS, WS, NY; Methodology: KS, WT, NS, PS, TT; Statistical analysis: KS, NS, TT ; Writing-original draft: KS, WT, NS, PS, NY, TT; Writing-review&editing: KS, WT, WS, NS, PS, NY, TT

Funding statement

This research received no specific grant from any funding agency in the public, commercial or not-for-profit sectors

A competing interests statement

There are no competing interests

Patient and public involvement

Patients and/or the public were not involved in the design, conduct, reporting, or dissemination of this research

Confidential: For Review Only

Patient consent form

Not applicable

Ethics approval

The protocol of this study was approved by the Ethics Committee of Rajavithi Hospital (Number 64237)

Data availability statement

The datasets used and/or analyzed during the current study are available from the corresponding author on reasonable request

Reference

1. Liu YC, Kuo RL, Shih SR. COVID-19: The first documented coronavirus pandemic in history. Biomed J. 2020;43(4):328-33.
2. Huang C, Wang Y, Li X, Ren L, Zhao J, Hu Y, et al. Clinical features of patients infected with 2019 novel coronavirus in Wuhan, China. Lancet. 2020;395(10223):497-506.
3. Xiao X, Newman C, Buesching CD, Macdonald DW, Zhou Z-M. Animal sales from Wuhan wet markets immediately prior to the COVID-19 pandemic. Scientific reports. 2021;11(1):11898.
4. A World Health Organization. WHO Director-General's opening remarks at the media briefing on COVID-19-11 March 2020. Available at: https://www.who.int/director general/speeches/detail/who-director-general-s-opening-remarks-at-the-media-briefing-on-covid-19--11-march-2020. Accessed December 29, 2021.
5. Ferguson N, Laydon D, Nedjati-Gilani G, Imai N, Ainslie K, Baguelin M, et al. Report 9: Impact of non-pharmaceutical interventions (NPIs) to reduce COVID-19 mortality and healthcare demand2020.
6. Eubank S, Eckstrand I, Lewis B, Venkatramanan S, Marathe M, Barrett CL. Commentary on Ferguson, et al., "Impact of Non-pharmaceutical Interventions (NPIs) to

Confidential: For Review Only

Reduce COVID-19 Mortality and Healthcare Demand". Bulletin of mathematical biology. 2020;82(4):52.

7. United Nations Educational, Scientific and Cultural Organization Institue for statistics : School closures and regional policies to mitigate learning loss due to COVID-19: a focus on the Asia-Pacific. Montreal, Quebec, Canada 2021.

8. The Economic Times. COVID-19 Impact : Screen time up by 100% for children. Available at: https://economictimes.indiatimes.com/industry/services/education/covid-19-impact-screen-time-up-by-100-for-children/articleshow/76383951.cms?from=mdr. Accessed December 29, 2021.

9. Twenge JM, Campbell WK. Associations between screen time and lower psychological well-being among children and adolescents: Evidence from a population-based study. Preventive Medicine Reports. 2018;12:271-83.

10. Ichhpujani P, Singh RB, Foulsham W, Thakur S, Lamba AS. Visual implications of digital device usage in school children: a cross-sectional study. BMC Ophthalmol. 2019;19(1):76.

11. Daum KM, Clore KA, Simms SS, Vesely JW, Wilczek DD, Spittle BM, et al. Productivity associated with visual status of computer users. Optometry (St Louis, Mo). 2004;75(1):33-47.

12. González-Pérez M, Susi R, Antona B, Barrio A, González E. The Computer-Vision Symptom Scale (CVSS17): development and initial validation. Invest Ophthalmol Vis Sci. 2014;55(7):4504-11.

13. Benedetto S, Drai-Zerbib V, Pedrotti M, Tissier G, Baccino T. E-readers and visual fatigue. PLoS One. 2013;8(12):e83676.

14. Seguí Mdel M, Cabrero-García J, Crespo A, Verdú J, Ronda E. A reliable and valid questionnaire was developed to measure computer vision syndrome at the workplace. J Clin Epidemiol. 2015;68(6):662-73.

15. Logaraj M, Madhupriya V, Hegde S. Computer vision syndrome and associated factors among medical and engineering students in chennai. Annals of medical and health sciences research. 2014;4(2):179-85.

16. Loebis R, Subakti Zulkarnain B, Zahra N. Correlation between the exposure time to mobile devices and the prevalence of evaporative dry eyes as one of the symptoms of

Confidential: For Review Only

computer vision syndrome among Senior High School students in East Java, Indonesia. J Basic Clin Physiol Pharmacol. 2021;32(4):541-5.

17. American Academy of Child & Adolescent Psychiatry. Screen Time and Children. Available at: https://www.aacap.org/AACAP/Families_and_Youth/Facts_for_Families/FFF-Guide/Children-And-Watching-TV054.aspx#:text=On%20average%2C%20children%20ages%208,use%20may%20lead%20to%20problems.Accessed April 8, 2022.

18. Lavin W, Taptagaporn S, Khruakhorn S, Kanchanaranya N. Prevalence and associated risk factors of digital eye strain among children in secondary schools in Pathumthani Province, Thailand. Journal of the Medical Association of Thailand. 2018;101:957-63.

19. Rosenfield M. Computer vision syndrome: a review of ocular causes and potential treatments. Ophthalmic Physiol Opt. 2011;31(5):502-15.

20. Iyer JV, Lee SY, Tong L. The dry eye disease activity log study. ScientificWorldJournal. 2012;2012:589875.

21. Blehm C, Vishnu S, Khattak A, Mitra S, Yee RW. Computer vision syndrome: a review. Surv Ophthalmol. 2005;50(3):253-62.

22. Al Tawil L, Aldokhayel S, Zeitouni L, Qadoumi T, Hussein S, Ahamed SS. Prevalence of self-reported computer vision syndrome symptoms and its associated factors among university students. Eur J Ophthalmol. 2020;30(1):189-95.

23. Parajuli BR, Koirala S, Bajracharya A. Computer vision syndrome: a rising problem during COVID-19 period amongst students and online workers. Journal of Patan Academy of Health Sciences. 2021;8(2):6-9.

24. Wang L, Wei X, Deng Y. Computer Vision Syndrome During SARS-CoV-2 Outbreak in University Students: A Comparison Between Online Courses and Classroom Lectures. Frontiers in public health. 2021;9:696036.

25. Ganne P, Najeeb S, Chaitanya G, Sharma A, Krishnappa NC. Digital Eye Strain Epidemic amid COVID-19 Pandemic - A Cross-sectional Survey. Ophthalmic epidemiology. 2021;28(4):285-92.

26. Li R, Ying B, Qian Y, Chen D, Li X, Zhu H, et al. Prevalence of Self-Reported Symptoms of Computer Vision Syndrome and Associated Risk Factors among School Students in China during the COVID-19 Pandemic. Ophthalmic Epidemiol. 2021:1-11.

Confidential: For Review Only

27. Mohan A, Sen P, Shah C, Jain E, Jain S. Prevalence and risk factor assessment of digital eye strain among children using online e-learning during the COVID-19 pandemic: Digital eye strain among kids (DESK study-1). Indian journal of ophthalmology. 2021;69(1):140-4.

28. Toh SH, Coenen P, Howie EK, Smith AJ, Mukherjee S, Mackey DA, et al. A prospective longitudinal study of mobile touch screen device use and musculoskeletal symptoms and visual health in adolescents. Applied ergonomics. 2020;85:103028.

29. Cantó-Sancho N, Sánchez-Brau M, Ivorra-Soler B, Seguí-Crespo M. Computer vision syndrome prevalence according to individual and video display terminal exposure characteristics in Spanish university students. International journal of clinical practice. 2021;75(3):e13681.

30. Straker L, Harris C, Joosten J, Howie EK. Mobile technology dominates school children's IT use in an advantaged school community and is associated with musculoskeletal and visual symptoms. Ergonomics. 2018;61(5):658-69.

31. Hu L, Yan Z, Ye T, Lu F, Xu P, Chen H. Differences in children and adolescents' ability of reporting two CVS-related visual problems. Ergonomics. 2013;56(10):1546-57.

32. Rempel D, Willms K, Anshel J, Jaschinski W, Sheedy J. The effects of visual display distance on eye accommodation, head posture, and vision and neck symptoms. Human factors. 2007;49(5):830-8.

33. Long J, Cheung R, Duong S, Paynter R, Asper L. Viewing distance and eyestrain symptoms with prolonged viewing of smartphones. Clinical & experimental optometry. 2017;100(2):133-7.

34. Teo C, Giffard P, Johnston V, Treleaven J. Computer vision symptoms in people with and without neck pain. Applied ergonomics. 2019;80:50-6.

35. Sen A, Richardson S. A study of computer-related upper limb discomfort and computer vision syndrome. Journal of human ergology. 2007;36(2):45-50.

36. Vasudevan B, Ciuffreda KJ, Gilmartin B. Sympathetic Inhibition of Accommodation after Sustained Nearwork in Subjects with Myopia and Emmetropia. Investigative Ophthalmology & Visual Science. 2009;50(1):114-20.

37. Sánchez-Valerio M, Mohamed-Noriega K, Zamora-Ginez I, Baez Duarte B, Vallejo-Ruiz V. Dry Eye Disease Association with Computer Exposure Time Among Subjects with Computer Vision Syndrome. Clinical Ophthalmology. 2020;Volume 14:4311-7.

Confidential: For Review Only

38. Calonge M, Pinto-Fraga J, González-García MJ, Enríquez-de-Salamanca A, López-de la Rosa A, Fernández I, et al. Effects of the External Environment on Dry Eye Disease. Int Ophthalmol Clin. 2017;57(2):23-40.
39. Zayed HAM, Saied SM, Younis EA, Atlam SA. Digital eye strain: prevalence and associated factors among information technology professionals, Egypt. Environ Sci Pollut Res Int. 2021;28(20):25187-95.
40. Mehra D, Galor A. Digital Screen Use and Dry Eye: A Review. Asia Pac J Ophthalmol (Phila). 2020;9(6):491-7.

Confidential: For Review Only

Table 1 Demographic characteristics of participants and usage of electronic devices before the pandemic (N=2,476)

Demographic characteristics	N (%)
Mean age (years) ± SD	15.52 ± 1.66 (range 10-19)
Female	1,606 (64.9%)
**Grade**	
Grade 4-6	11 (0.4%)
Grade 7-9	781 (31.6%)
Grade 10-12	1684 (68%)
**Refractive status**	
Emmetropia	1180 (47.7%)
Myopia	1055 (42.6%)
Hyperopia	241 (9.7%)
**Glasses and Contact lens use**	
None	1180 (47.7%)
Glasses for myopia	784 (31.7%)
Glasses for hyperopia	186 (7.5%)
CL for myopia	151 (6.1%)
CL for hyperopia	47 (1.9%)
Glasses and CL for myopia	120 (4.8%)
Glasses and CL for hyperopia	8 (0.3%)
**Most commonly-used digital devices (pre-covid)**	
Mobile phone	1341 (54.2%)
Tablet	454 (18.3%)
Computer desktop	436 (17.6%)
Computer Laptop	216 (8.7%)
Television	29 (1.2%)
**Frequency of eye check-up**	
None	1054 (42.6%)
Once every 2 years	504 (20.4%)
Once a year	715 (28.9%)
Twice a year	203 (8.2%)

Confidential: For Review Only

Table 2 Comparison between duration of digital device usage per day before and during COVID-19 lockdown.(N=2,476)

Duration of digital device usage per day	Before COVID-19	During COVID-19
≤ 2h	201 (8.1%)	67 (2.7%)
3-4 h	596 (24.1%)	271 (21.9%)
5-6 h	648 (26.2%)	502 (20.3%)
7-8 h	492 (19.9%)	1109 (44.8%)
9-10 h	401 (16.2%)	480 (19.4%)
11-12 h	49 (1.9%)	16 (0.7%)
> 12 h	89 (3.6%)	31 (1.3%)
**Number of hours spent per day (mean ± SD)**	6.13 ± 2.8	10.53 ± 2.99

Confidential: For Review Only

Table 3 The usage of electronic devices in online learning during covid-19 lockdown (N=2,476)

Demographic characteristics	N (%)
**Total hours of online learning**	
≤ 2h	67 (2.7%)
3-4 h	271 (11%)
5-6 h	502 (20.3%)
7-8 h	1109 (44.8%)
9-10 h	480 (19.4%)
> 10 h	47 (1.8%)
**Device used for online learning**	
Single device	1486 (60%)
Multiple device	990 (40%)
Mobile phone	1174 (47.4%)
Tablet	1095 (44.2%)
Computer desktop	965 (39%)
Computer Laptop	691 (27.9%)
Television	27 (1.1%)
**Environment**	
Fan	1272 (51.4%)
Air-conditioning	1203 (48.6%)
**Protective instrument**	
None	413 (16.7%)
Blue-coated glasses	1172 (47.3%)
Blue coated film on digital devices	680 (27.5%)
Both blue-coated glasses and film on digital device	211 (8.5%)
**Frequency of eye rest**	
Never	371 (15%)
Every 15 minutes	596 (24.1%)
Every 30 minutes	431 (17.4%)
Every 45 minutes	304 (12.3%)
Every 1 hours	427 (17.2%)
Every ≥ 1 hour	347 (14%)
**Distance of digital devices from eyes during online learning**	
< 40 cm	943 (38.1%)
40-80 cm	1260 (50.9%)
> 80 cm	273 (11%)
**Back pain**	1880 (75.9%)
**Neck pain**	1687 (68.1%)
**Activity when resting**	
Close eyes	1144 (46.2%)
Sleep	1230 (49.7%)
Look out	822 (33.2%)
Play games	568 (22.9%)
Use artificial tears	271 (10.9%)

Confidential: For Review Only

Table 4 Multiple logistic regression showing the associated risk factors of CVS

**Factor**	**Univariate analysis**			**Multiple analysis**		
	Crude OR	95%CI	P-value	Adjusted OR	95%CI	P-value
Age ≤ 15	2.34	1.63-3.36	<0.001	2.17	1.36-3.45	0.010
Female	1.73	1.34-2.23	<0.001	1.26	0.90-1.75	0.178
Overall digital usage > 6 hr	7.41	5.52-9.96	<0.001	1.91	1.13-3.23	0.016
Online learning > 5 hr	7.99	6.07-10.53	<0.001	4.99	3.08-8.12	<0.001
Refractive error	1.35	1-02-1.79	0.035	2.89	1.83-4.54	<0.001
**Refractive error**						
Myopia	3.21	2.31-4.44	<0.001	2.11	1.24-3.32	<0.001
Emmetropia	3.19	2.21-4.60	<0.001	2.09	2.14-3.47	<0.001
Hyperopia	Ref			Ref		
Fan	2.55	1.86-3.48	<0.001	1.20	0.81-1.81	0.362
Non-Protective device used	1.82	1.19-2.79	0.006	1.07	0.66-1.73	0.793
**Protective device**						
Both	4.62	2.90-7.38	<0.001	0.66	0.39-1.11	0.118
Blue-coated glasses	3.36	2.52-4.49	<0.001	1.19	0.71-1.99	0.508
Blue-coated film	Ref			Ref		
Rest of over 45 mins	2.59	2.01-3.34	<0.001	1.02	0.7-1.48	0.935
Other digital device used > 2 hr	2.22	1.73-2.85	<0.001	0.89	0.61-1.28	0.520
Distance from device < 40 cm	2.42	1.78-3.30	<0.001	1.07	0.71-1.63	0.743
Back pain	2.84	2.12-3.80	<0.001	2.06	1.32-3.22	0.001
Neck pain	2.59	1.94-3.46	<0.001	2.64	1.89-3.70	<0.001
Multiple digital devices used	3.60	2.76-4.70	<0.001	2.15	1.04-4.43	0.038
Non-use of artificial tears	0.96	0.70-1.30	0.77	1.34	0.91-1.98	0.133
Close eye during online learning	1.69	1.32-2.17	<0.001	1.12	0.80-1.55	0.508
Sleep during online learning	2.12	1.64-2.74	<0.001	1.29	0.93-1.79	0.135
Laptop computers preference	2.36	1.75-3.19	<0.001	0.83	0.48-1.42	0.494
Tablet preference	2.27	1.77-2.92	<0.001	0.86	0.51-1.45	0.568
Television screen preference	1.49	0.49-4.55	0.49	1.067	0.30-3.75	0.920
Computer desktop preference	1.37	1.07-1.77	0.016	1.00	0.61-1.66	0.987
Mobile phone preference	1.34	1.03-1.69	0.031	0.81	0.50-1.31	0.395

Confidential: For Review Only

Figure 1 The mean number of hours per day of online learning and total hours per day of digital device usage according to CVS severity.

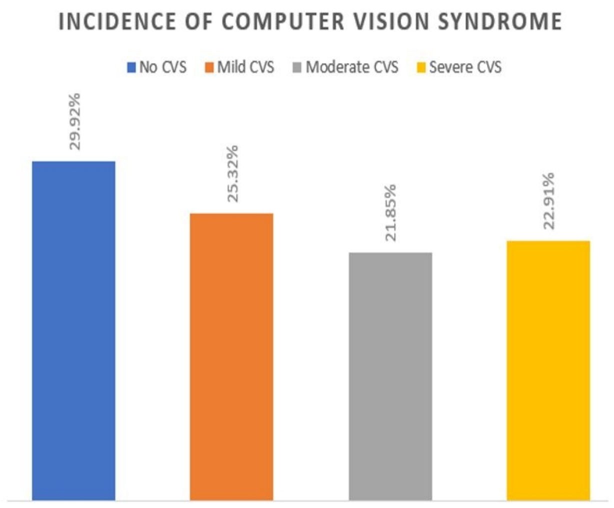

	No CVS (n=741)	Mild CVS (n=627)	Moderate CVS (n=541)	Severe CVS (n=567)	P-Value
Mean number of hours per day of online learning (hours) ± SD	6.16 ± 2.26	7.23 ± 2.01	7.28 ± 1.78	7.69 ± 1.74	< 0.001
Mean hours per day of total digital device usage (hours) ± SD	9.45 ± 3.38	10.77 ± 2.93	10.89 ± 2.57	11.33 ± 2.54	< 0.001

101x91mm (300 x 300 DPI)

Figure 2 The percentage of frequency and severity of different eye symptoms of CVS in student

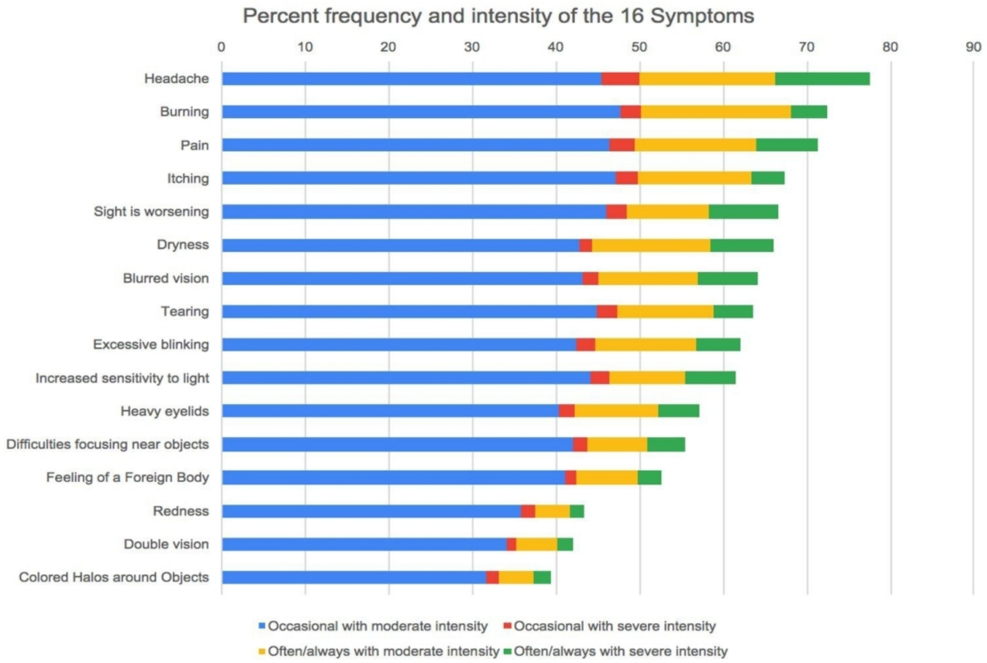

128x91mm (300 x 300 DPI)

Effects of digital devices and online learning on computer vision syndrome in students during the COVID-19 era

<u>Survey</u>

The purpose of this survey: This survey aims to gather information from students of different year groups about what visual symptoms they experience and how well they protect their eyes during the period of online learning, since it is compulsory for students to sit in front of their screens for a long period of time. This results of this survey will then be analyzed, conclusion will be drawn and the results will then be incorporated as one of the research findings.

General overview of the survey:
First section of the survey - 8 questions
Second section of the survey - 8 questions
Third section of the survey - 18-36 questions

It will take approximately 2 minutes to fill out the survey!

Please note that the survey will be anonymous. The information will be kept confidential and will only be used for this research.

Informed consent

1. Do you voluntarily agree to complete this survey?
   - I voluntarily agreed to complete this survey

Section 1 of the survey: General information

1. Age

   ____

2. Gender
   - Male
   - Female
3. Grade level
   - Grade 1-3/Year 2-4
   - Grade 4-6/Year 5-7
   - Grade 7-9/Year 8-10
   - Grade 10-12/Year 11-13
4. Before the period of online learning, how many hours per day do you spend on average using electronic devices?
   - 0 hour
   - 1 hour
   - 2 hours
   - 3 hours
   - 4 hours
   - 5 hours
   - 6 hours
   - 7 hours

Confidential: For Review Only

- 8 hours
- 9 hours
- 10 hours
- 11 hours
- 12 hours
- Other……………..

5. Before the period of online learning, what electronic devices did you use most often?
   - Tablet (iPad)
   - Mobile Phone
   - Computer/Desktop
   - Laptop
   - Television
   - Other
6. Do you wear any of these? (Can tick more than one box)
   - Glasses for short-sightedness
   - Glasses for long-sightedness
   - Contact lenses for short-sightedness
   - Contact lenses for long-sightedness

7. How often do you have an eye check-up?
   - Never
   - Once every two years
   - Once a year
   - Twice a year
   - Other

Section 2 of the Survey

7. What electronic devices do you use for online learning?
   - Tablet (iPad)
   - Mobile Phone
   - Computer/Desktop
   - Laptop
   - Television
   - Other
8. How many hours per day do you spent learning online?
   - 1 hour
   - 2 hours
   - 3 hours
   - 4 hours
   - 5 hours
   - 6 hours
   - 7 hours
   - 8 hours
   - 9 hours
   - 10 hours
   - 11 hours

Confidential: For Review Only

- 12 hours
- Other…………….

9. What is the environment of your room when you learn online? Do you use a fan or an air conditioner?
    - Fan
    - Air conditioner
    - Other
10. Do you use any equipment to help protect your eyes?
    - Glasses
    - Films for computer/tablets/phone screens
11. How often do you give your eyes a rest during online classes?
    - Never
    - Every 15 minutes
    - Every 30 minutes
    - Every 45 minutes
    - Every 1 hour
    - Every 2 hours
    - Other
12. What do you do when you rest your eyes?
    - Close your eyes
    - Look out of the window as far as you could
    - Play games on your device
    - Sleep
    - Artificial tear
    - Other
13. Apart from using electronic devices for online learning, how much time do you spend using electronic devices for other activities?
    - 1 hour
    - 2 hours
    - 3 hours
    - 4 hours
    - 5 hours
    - 6 hours
    - 7 hours
    - 8 hours
    - 9 hours
    - 10 hours
    - 11 hours
    - 12 hours
    - Other…………….
14. How far apart do you position your devices from your eyes?
    - 20-40 cm
    - 40-60 cm
    - 60-80 cm
    - 80-100 cm
    - more than 100 cm

Confidential: For Review Only

Section 3 of the survey:
This section will be the computer vision syndrome questionnaire.

The first question will be related to the frequency of the symptom, how often the symptom occurs:
Never = the symptom does not occur at all
Occasionally = Sporadic episodes or once a week
Often or always = 2 or 3 times a week or almost everyday

NOTE: If you have answered "Never" for the first question, you will not be asked about the intensity, but if you answered "Occasionally" or "Often or always", you will be required to answer the question about intensity.

The second question will be related to the intensity of the symptom. You are required to choose between "moderate" or "Intense" for each of the symptoms.

15. When you learn online, do you experience the symptom of eye burning?
    - Never (the symptom does not occur)
    - Occasionally (once a week)
    - Often or always (2 or 3 times a week or almost everyday)
16. What is the intensity of the symptom? (If you answered never, do not answer this question)
    - Moderate
    - Intense
17. When you learn online, do you experience the symptom of eye itching?
    - Never (the symptom does not occur)
    - Occasionally (once a week)
    - Often or always (2 or 3 times a week or almost everyday)
18. What is the intensity of the symptom? (If you answered never, do not answer this question)
    - Moderate
    - Intense
19. When you learn online, have you ever feel like there's foreign body in your eyes?
    - Never (the symptom does not occur)
    - Occasionally (once a week)
    - Often or always (2 or 3 times a week or almost everyday)
20. What is the intensity of the symptom? (If you answered never, do not answer this question)
    - Moderate
    - Intense
21. When you learn online, do you experience the symptom of tearing?
    - Never (the symptom does not occur)
    - Occasionally (once a week)
    - Often or always (2 or 3 times a week or almost everyday)
22. What is the intensity of the symptom? (If you answered never, do not answer this question)
    - Moderate

Confidential: For Review Only

- Intense

23. When you learn online, do you experience the symptom of excessive blinking?
    - Never (the symptom does not occur)
    - Occasionally (once a week)
    - Often or always (2 or 3 times a week or almost everyday)
24. What is the intensity of the symptom? (If you answered never, do not answer this question)
    - Moderate
    - Intense
25. When you learn online, do you experience the symptom of eye redness?
    - Never (the symptom does not occur)
    - Occasionally (once a week)
    - Often or always (2 or 3 times a week or almost everyday)
26. What is the intensity of the symptom? (If you answered never, do not answer this question)
    - Moderate
    - Intense
27. When you learn online, do you experience the symptom of eye pain?
    - Never (the symptom does not occur)
    - Occasionally (once a week)
    - Often or always (2 or 3 times a week or almost everyday)
28. What is the intensity of the symptom? (If you answered never, do not answer this question)
    - Moderate
    - Intense
29. When you learn online, do you experience the symptom of heavy eyelids?
    - Never (the symptom does not occur)
    - Occasionally (once a week)
    - Often or always (2 or 3 times a week or almost everyday)
30. What is the intensity of the symptom? (If you answered never, do not answer this question)
    - Moderate
    - Intense
31. When you learn online, do you experience the symptom of eye dryness?
    - Never (the symptom does not occur)
    - Occasionally (once a week)
    - Often or always (2 or 3 times a week or almost everyday)
32. What is the intensity of the symptom? (If you answered never, do not answer this question)
    - Moderate
    - Intense
33. When you learn online, do you experience the symptom of blurred vision?
    - Never (the symptom does not occur)
    - Occasionally (once a week)
    - Often or always (2 or 3 times a week or almost everyday)
34. What is the intensity of the symptom? (If you answered never, do not answer this question)

Confidential: For Review Only

- Moderate
- Intense

35. When you learn online, do you experience the symptom of double vision?
    - Never (the symptom does not occur)
    - Occasionally (once a week)
    - Often or always (2 or 3 times a week or almost everyday)
36. What is the intensity of the symptom? (If you answered never, do not answer this question)
    - Moderate
    - Intense
37. When you learn online, how often do you experience difficulties focusing near objects?
    - Never (the symptom does not occur)
    - Occasionally (once a week)
    - Often or always (2 or 3 times a week or almost everyday)
38. What is the intensity of the symptom? (If you answered never, do not answer this question)
    - Moderate
    - Intense
39. When you learn online, do you experience the symptom of increased sensitivity to light?
    - Never (the symptom does not occur)
    - Occasionally (once a week)
    - Often or always (2 or 3 times a week or almost everyday)
40. What is the intensity of the symptom? (If you answered never, do not answer this question)
    - Moderate
    - Intense
41. When you learn online, do you experience the symptom of colored halos around objects?
    - Never (the symptom does not occur)
    - Occasionally (once a week)
    - Often or always (2 or 3 times a week or almost everyday)
42. What is the intensity of the symptom? (If you answered never, do not answer this question)
    - Moderate
    - Intense
43. When you learn online, do you experience the feeling that sight is worsening?
    - Never (the symptom does not occur)
    - Occasionally (once a week)
    - Often or always (2 or 3 times a week or almost everyday)
44. What is the intensity of the symptom? (If you answered never, do not answer this question)
    - Moderate
    - Intense
45. When you learn online, how often do you have a headache?
    - Never (the symptom does not occur)

Confidential: For Review Only

- Occasionally (once a week)
- Often or always (2 or 3 times a week or almost everyday)

46. What is the intensity of the symptom? (If you answered never, do not answer this question)
    - Moderate
    - Intense
47. When you learn online, how often do you experience back pain?
    - Never (the symptom does not occur)
    - Occasionally (once a week)
    - Often or always (2 or 3 times a week or almost everyday)
48. What is the intensity of the symptom? (If you answered never, do not answer this question)
    - Moderate
    - Intense
49. When you learn online, how often do you experience neck pain?
    - Never (the symptom does not occur)
    - Occasionally (once a week)
    - Often or always (2 or 3 times a week or almost everyday)
50. What is the intensity of the symptom? (If you answered never, do not answer this question)
    - Never (the symptom does not occur)
    - Occasionally (once a week)
    - Often or always (2 or 3 times a week or almost everyday)

Thank you for completing this survey!

Confidential: For Review Only
